# Mutations of the SARS-CoV-2 Spike Glycoprotein Detected in Cats and Their Effect on Its Structure and Function

**DOI:** 10.3389/fcimb.2022.875123

**Published:** 2022-06-01

**Authors:** Mervat E. Hamdy, Ayman H. El-Deeb, Naglaa M. Hagag, Momtaz A. Shahein, Osama Alaidi, Hussein A. Hussein

**Affiliations:** ^1^ Genome Research Unit, Animal Health Research Institute, Agriculture Research Centre, Giza, Egypt; ^2^ Department of Virology, Faculty of Veterinary Medicine, Cairo University, Giza, Egypt; ^3^ Department of Virology, Faculty of Veterinary Medicine, King Salman International University, South Sinai, Egypt; ^4^ Department of Virology, Animal Health Research Institute, Agriculture Research Centre, Giza, Egypt; ^5^ Department of Research and Development, Biocomplexity for Research and Consulting, Cairo, Egypt; ^6^ Department of Pharmaceutical Sciences, University of Tennessee Health Science Center, Memphis, TN, United States

**Keywords:** SARS-CoV-2, spike, mutations, ACE2, modeling, cats

## Abstract

The high frequency of Severe Acute Respiratory Syndrome Coronavirus-2 (SARS-CoV-2) mutations and homology of the Angiotensin-Converting Enzyme-2 (ACE2) cell receptors in various hosts help the virus transcend species barriers. In this study, we investigated the mutations of the SARS-CoV-2 spike glycoprotein detected in cats and their effect on its structure and function. Interestingly, some of these mutations are reported here in cats for the first time. Structural analysis showed seven residue substitutions in the spike glycoprotein. Four of the detected mutations are located on the spike surface, which are critical interaction points for neutralizing antibodies. Furthermore, three of the reported mutations could facilitate viral binding to the ACE2 host receptor, influence S1/S2 cleavage, destabilize the β-hairpin structure of the S2 and enhance viral infectivity. Structural modeling and phylogenic analysis of the ACE2 receptor provided an indication of the binding capacity of the virus to the specific cell receptors of different species and hosts. The presented work highlights the effects of the residue substitutions on viral evasion, infectivity and possibility of SARS-CoV-2 spillover between humans and cats. In addition, the work paves the way for in-depth molecular investigation into the relationship between SARS-CoV-2 receptor binding and host susceptibility.

## Introduction

Severe acute respiratory syndrome coronavirus 2 (SARS-CoV-2), which triggered the Coronavirus Disease 2019 (COVID-19) outbreak, has caused enormous socioeconomic losses (Zhou et al., 2020). This virus is a member of the *Nidovirales* order, the *Coronaviridae* family and the *Coronavirinae* subfamily in the *Betacoronavirus* genus. SARS-CoV-2 is a spherical pleomorphic virus (50–140 nm in size). It is an enveloped virus with large, 20 nm-long, club-shaped peplomers that form a crown appearance. It has a helical nucleocapsid with the largest positive sense RNA (29,903 bases) (Lu et al., 2020). There are two protein groups in SARS-CoV-2 (structural and non-structural). The structural proteins include the spike glycoprotein (S), the nucleocapsid (N), the membrane protein (M) and the envelope protein (E) (Naqvi et al., 2020); whereas the non-structural proteins include the protease (NSP3 and NSP5), the RNA-dependent RNA polymerase protein (NSP12) (Begum et al., 2020), helicase (NSP13), endoribonuclease (NSP15) and other NSPs (NSP1, NSP2, NSP4, NSP6, NSP7, NSP8, NSP9, NSP10, NSP11, NSP14 and NSP16) that associated with immune suppression, viral replication and transcription (Chan et al., 2020).

**Figure 1:**
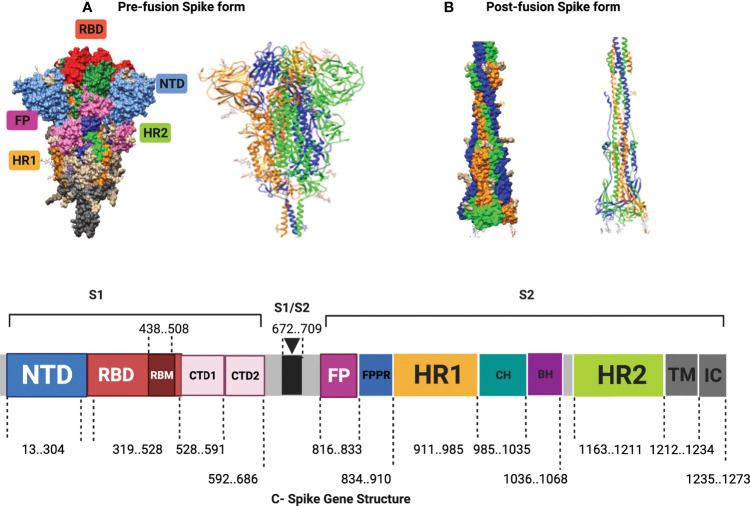
The structure of the spike glycoprotein and its coding gene. **(A)** shows a schematic surface view of the spike ectodomain and its constituting domains and regions. **(B)** illustrates the structure of the native spike glycoprotein in its pre-fusion and post-fusion states as determined by Cryo-EM (PDB ID 6xr8 and 6xra, respectively) (Cai et al., 2020) in which the spike trimer is composed of three interacting chains or protomers depicted here in distinct colors. **(C)** demonstrates the detailed structure of the spike gene with the locations of the different regions shown according to the Wuhan strain (Reference Sequence NC_045512.2, Gene ID: 43740568) (Guruprasad, 2021; NCBI, 2021). The spike glycoprotein is composed of 2 subunits (S1 and S2). The S1 subunit includes the N-terminal domain (NTD), the receptor binding domain (RBD) which carries the receptor-binding motif (RBM), and two structurally conserved subdomains (the C- terminal domains 1 (CTD1) and the C- terminal domains 2 (CTD2). The S2 subunit contains the N-terminal hydrophobic fusion peptide (FP), the fusion peptide proximal region (FPPR), the heptad repeat 1 (HR1) motif, the central helix region (CH), the β-hairpin region, the connector domain (CD)and the heptad repeat 2 (HR2) motif. These are followed by the transmembrane region (TM) and the intracellular regions (IC) (Guruprasad, 2021; NCBI, 2021). The figure was assembled with Biorender website (Biorender, 2021).

The SARS CoV-2 spike glycoprotein initiates a viral infection through its attachment to angiotensin-converting enzyme 2 (ACE2) cell receptors, which are distributed widely in different organs, including oro/nasopharyngeal mucosae, the lungs, gastrointestinal tract (GIT) mucosae, the brain, kidneys and the liver (Hamming et al., 2004). The spike glycoprotein is a trimeric fusion molecule (as illustrated in **Figure 1**), and its corresponding gene consists of 3,822 nucleotides (21,563–25,384) that encode 1,273 amino acids. The spike glycoprotein is composed of two main subunits, an amino (N)-terminal S1 subunit and a carboxyl (C)-terminal S2 subunit, that are cleaved at the furin cleavage site (S1/S2 cleavage region) (Chan et al., 2020). The detailed molecular structure of the spike gene with detailed regions and locations is illustrated in **Figure 1** and tabulated in **Supplementary Table S1**, according to the Wuhan strain (Reference Sequence NC_045512.2, GeneID: 43740568) (Guruprasad, 2021; NCBI, 2021). The S1 subunit interacts with the ACE2 receptor subsequently promoting the viral infection (Hoffmann et al., 2020), and this interaction has an impact on both cross-species and human-to-human transmissions (Lu et al., 2015; Wan et al., 2020). The S1 subunit switches between a standing up receptor-binding domain (RBD) (e.g., one or two RBD-up conformers), amenable to ACE2 binding, and closed-down positions, undergoing spontaneous conformational transitions between ensembles of closed and open receptor accessible forms (Wan et al., 2020; Wang et al., 2020). In contrast with the buried S2 subunit, the S1 subunit domains are located on the surface of the spike glycoprotein, effectively protecting the fusion apparatus (Shang et al., 2020). Upon proteolytic activation at the S1/S2 region, the S1 subunit is dissociated from the S2 (Shang et al., 2020). Following the latter dissociation event, the S2 subunit undergoes a series of enormous structural rearrangements in order to orchestrate the fusion of cellular and viral membranes (Duan et al., 2020; Shang et al., 2020; Wan et al., 2020; Wang et al., 2020).

SARS-CoV-2 has evolved at a rate of approximately 1.5–3.3 × 10^−3^/per site/year (Chaw et al., 2020). Consequently, residue substitutions may affect viral infectivity, pathogenesis, transmission and viral virulence (Shafique et al., 2020). The high mutation rate and recombination frequency is a significant dilemma that may have a role in cross-species transmission (Elaswad et al., 2020). SARS-CoV-2, which has a bat precursor, evokes many questions regarding its natural origin, its transmissibility to different animal hosts and the possible incidence of animal viral reservoirs (Elaswad et al., 2020). Many experiments have investigated the susceptibility of different species to SARS-CoV-2 infection, including non-human primates, ferrets, cats, dogs, minks, tigers, mice and pigs (Hobbs and Reid, 2021). Furthermore, a case of zoonotic transmission from minks to farm workmen in Denmark was investigated (Prince et al., 2021).

**Figure 2:**
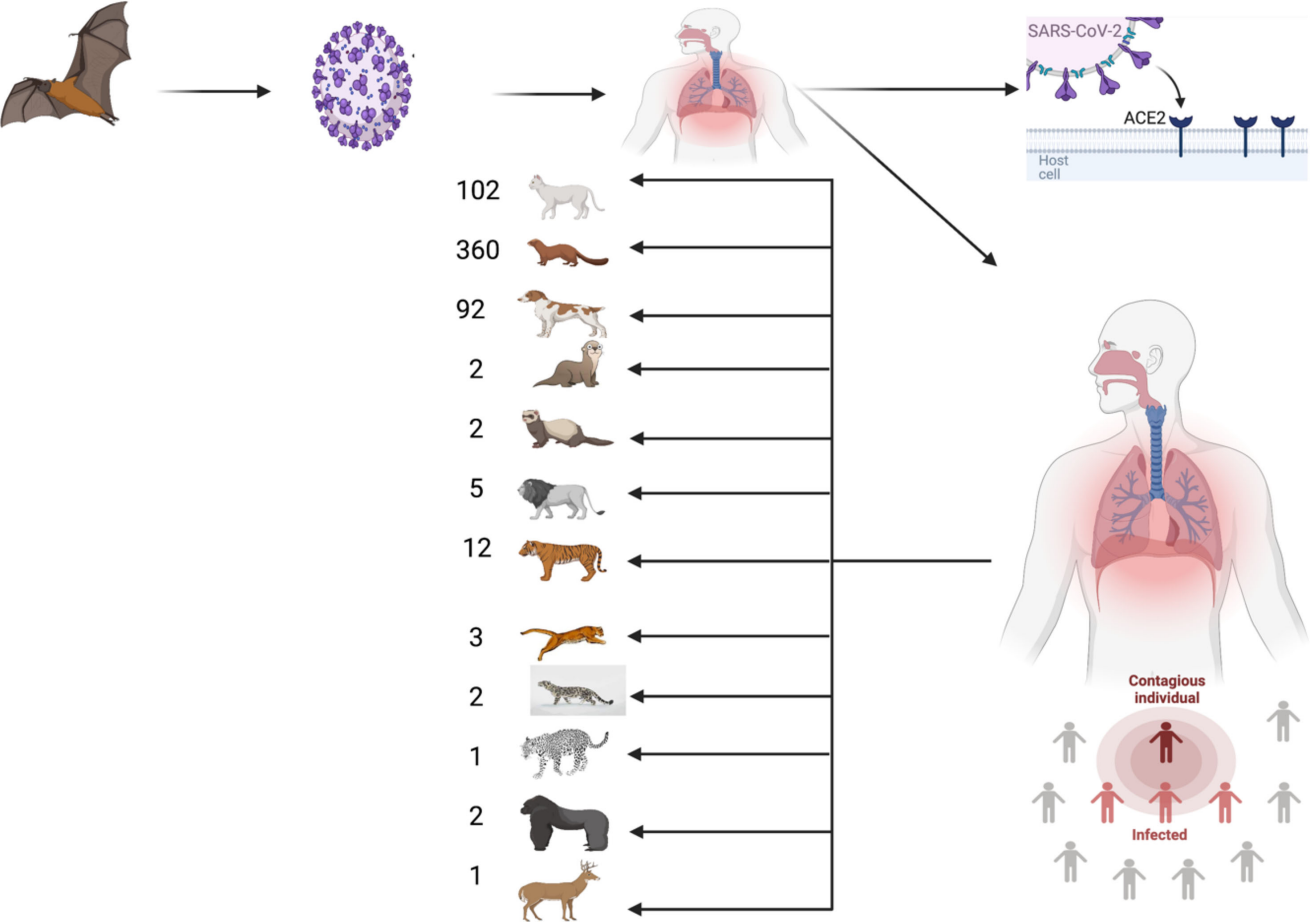
COVID-19 outbreaks in animals. The figure illustrates the number of outbreaks reported worldwide by species according to the fifth OIE report (till 30 September 2021) (OIE, 2021). The figure was assembled with Biorender website (Biorender, 2021).

The cross-species transmission of coronaviruses and their ability to jump species barriers have been reported. Moreover, many research papers have investigated the factors that may strengthen the capability of coronaviruses to overcome species barriers and increase their virulence (Elaswad et al., 2020). These features are illustrated not only by the high mutation rates of coronaviruses (Su et al., 2016), but also by the large RNA genome of these viruses that allows the occurrence of recombination and mutation, resulting in the emergence of new coronaviruses (Ye et al., 2020). Despite the importance of these features, the interaction ability of coronaviruses with different ACE2 receptors has become the dominant and most important virulence factor of these viruses (Bolles et al., 2011). According to the fifth report of the Office International des Épizooties (OIE) regarding the SARS-CoV-2 situation up until the end of September 2021, 584 outbreaks in animals (mostly in minks, cats and dogs) were reported globally, affecting 12 species in 30 countries (as demonstrated in **Figure 2**) (OIE, 2021). Therefore, the OIE strongly encourages the reporting of SARS-CoV-2 occurrence in animals (OIE, 2021).

Furthermore, in our recent study, we reported SARS-CoV-2 spillover between humans and pet animals in Egypt in 30.3% of cats (10/33) and 24.2% of dogs (8/33), as evidenced by real-time reverse transcriptase polymerase chain reaction (rRT-PCR) (Mervat et al., 2021). Moreover, thrombocytopenia, lymphocytopenia and high levels of ferritin, C-reactive protein and D-dimers were reported in hematology and clinical studies (Mervat et al., 2021).

In order to further investigate such interspecies transmission, in this study, we conducted a full spike gene sequencing of SARS-CoV-2 detected in four cats, and their treating veterinarian. Samples selection was based on the severity of clinical signs. Sequencing was combined with computational analysis, including structural visualization and homology modeling of the glycoprotein to investigate the mutations and their effect on its structure and function. Structural modelling of the ACE2 receptor in different species was conducted to study the host susceptibility to SARS-CoV-2 infection.

## Materials and Methods

### Ethical Approval

The study was conducted according to ethical guidelines and approved by the Ethics Committee of Animal Health Research Institute (protocol code 11429 and date of approval 12/2020).

### Sample Collection and Preparation

Swabs from oro/nasopharyngeal mucosa were collected from four cats, (three of these cats had severe respiratory signs and one of them were a symptomatic), that were in contact with COVID-19 positive owners. Additionally, a sample was taken from a veterinarian who had been in contact with these cats in his clinic and he was asymptomatic. These samples were collected in December 2020, March 2021 and July 2021. The swabs were deposited into sterile tubes containing 2 ml of autoclaved phosphate buffered saline (PBS). The latter samples were collected independently from different houses and represent independent outbreaks. The possibility of SARS-CoV-2 spillover was based on infection’s chronology, symptoms onset and case history, as owners were infected with SARS-CoV-2 before symptoms appear on their animals, and hence they likely have transmitted the virus to their companion animals.

### Nucleic Acid Extraction and Molecular Diagnosis

According to the manufacturer’s instructions, viral ribonucleic acid (RNA) was extracted automatically using a MagPurix extractor (Labgene scientific, Switzerland). This was followed by a SARS-CoV-2 screening of the extracted RNA with rRT-PCR using a transgenbiotech multiplex SARS-CoV-2 diagnostic kit (Cat no. DV101) that detects two genes (nucleocapsid and ORF1ab genes). The cycling protocol of the rRT-PCR was composed of 10 minutes at 50°C and 30 seconds at 95°C, followed by 40 cycles of 5 seconds at 95°C then 30 seconds at 60°C.

### Full-Length Spike Gene Amplification

The full-length spike gene of the spike glycoprotein was amplified for the rRT-PCR positive samples using conventional RT-PCR utilizing spike gene-specific primers, as reported previously (Ren et al., 2020) and tabulated in (**Supplementary Table S2**). Briefly, the RT-PCR reaction was set up using an RT-PCR kit (PrimeScript, Takara, California, USA). The cycling conditions were as follows: a cycle of 30 minutes at 50°C for the activation of reverse transcriptase (RT); then 2 minutes at 95°C for the deactivation of the RT; followed by 40 cycles of 95°C for 30 seconds, 50°C for 30 seconds and 72°C for 1.5 minutes; with a final extension cycle of 72°C for 10 minutes.

### Sequencing and Phylogenetics

According to the manufacturer’s recommendations, positive DNA bands with the expected sizes were cut and purified using a gel extraction kit (QIAquick, Qiagen). The BigDye Terminator 3.1 Cycle Sequencing Kit was used to sequence the purified products (Applied Biosystems, USA). Next, the Centri-Sep Purification Kit (Applied Biosystem) was used to purify the sequencing reactions. The purified sequence products were then decoded using the 3500 Genetic Analyzer (Applied Biosystems). The BioEdit Sequence Alignment Editor 7.2.5 (Hall, 1999) was used to prepare protein multiple sequence alignments. MEGA7 software was used to create a midpoint rooted maximum likelihood phylogenetic tree with 1000 bootstrap replicates to verify the tree (Kumar et al., 2016). This tree depends on the Jones–Taylor–Thornton (JTT) matrix-based model (Jones et al., 1992) and the tree is illustrated with the highest likelihood log value (–11894.02) in which related taxa and closely related branches are clustered together. The initial tree for the heuristic search was automatically generated with application of the neighbor join and BioNJ algorithms to a matrix of pairwise distances predicted using the JTT model, then the topology with the highest log likelihood value was chosen. The branch lengths were calculated by the number of residue substitutions per site, and the tree is depicted to scale. MEGA7 software was used to display the evolutionary analysis (Kumar et al., 2016).

### Structural Visualization and Obtaining a Homology Model of the Spike Mutant

Cartoon illustrations were generated using UCSF Chimera molecular visualization software (Pettersen et al.,2004). Atomic coordinates from PDB (ID 6xr8 and 6xra (Cai et al., 2020) were used to represent the native structures for the pre-fusion and post-fusion (S2) spike glycoprotein ectodomain trimer, respectively; whereas atomic coordinates from PDB ID *7krq* and *7krr* (Zhang J et al., 2021) were used to represent the (D614G) closed and one RBD-up open conformers, respectively. The homology model of the spike glycoprotein chains (i.e., trimer) for the sequence presented in this study (hCoV-19/Cat-Kitten/Egypt/AHRI/July/2021) was generated using the MODELLER software (Eswar et al., 2003), with the (D614G) model (PDB ID *7krr*, which is in the open state with one RBD-up) as a template. The sequences were aligned using the MUSCLE program (Edgar, 2004), and the columns corresponding to missing residues in the template model were removed before submission to the MODELLER program.

### Homology Modeling of the ACE2 Receptors in Various Species and Their Phylogenetic Analysis

To be able to compare the receptors from various animal species, a BLASTP (Altschul et al., 1990) search was performed using the reference sequence for human ACE2 receptor against the NCBI Protein Reference Sequence database (Refseq_protein). Over 1200 significant hits were found. Sequences of the best 249 hits (i.e., those with the highest sequence identity) were retrieved and aligned using the COBALT multiple sequence alignment tool (Papadopoulos and Agarwala, 2007) through the NCBI server. Throughout this paper, all residue numbers for the receptor refer to the human ACE2 isoform 1 protein reference sequence (accession: NP_001358344.1). The alignment blocks of interest were extracted using the extractalign tool in the EMBOSS package (Rice et al., 2000). The alignment blocks for the main residues (or regions) that were in contact with the viral RBD in the structure of the RBD-ACE2 complex were used to generate sequence logos using the WebLogo Server (Schneider and Stephens, 1990; Crooks et al., 2004), namely alignment blocks incorporating residues 30–41, residues 82–84 and residues 353–357 were used. The residues coding for the signal peptide (residues 1–17) were removed from the alignment. The alignment blocks of residues 19–90 and of 324–393 were joint, and were then used for the construction of the ACE2 phylogenetic tree.

To obtain further structural insights into the differential binding capacity of the various host species receptors with the unmutated viral RBD, we built homology models of the receptor-RBD complexes of five distinct species. Specifically, models from cat, dog, ermine, ferret and chicken ACE2 receptor sequences were built. Homology models were built (also using MODELLER software) for the peptidase domains (PD domain, residues 18–614) of the ACE2 sequences of each species with a fixed COVID-19 RBD domain. For simplicity, glycosylation sites, which are far from the receptor-binding interface, as well as bound water molecules in the template structures were ignored. However, the Zinc atoms were included in the built homology models as they may be essential for stabilizing the structures. The receptor-RBD complexes (PDB IDs 6LZG and 6M17) were used as templates. A model for the human ACE2 isoform 1 protein reference sequence was also built and used as a control. For each species-specific receptor-RBD complex, the model with the best score using MODELLER was used in the subsequent steps. Hydrogen atoms were added to each model assuming a neutral pH = 7.0, and the structure was then minimized using all atom Amber14 force fields (“amber14-all”) (Maier et al., 2015) in vacuum to remove any clashes. The energy minimization was performed using OpenMM Python API (Eastman et al). Isosurface representations and electrostatic (Coulombic) potential coloring of the isosurfaces were performed using Chimera (Pettersen et al., 2004).

Next, a phylogenetic tree was constructed for all residues at the binding interface between the ACE2 and the viral RBD (19–90 and 324–393) in different species. MEGA7 software was used to create a midpoint rooted maximum likelihood tree with 1000 bootstrap replicates to verify this tree (Kumar et al., 2016). A maximum likelihood technique depending on the JTT matrix-based model was used (Jones et al., 1992) with the highest likelihood log value (–1606.65). The related taxa and closed branches were clustered together. The initial tree for the heuristic search was automatically generated with the application of the neighbor join and BioNJ algorithms to a matrix of pairwise distances predicted using the JTT model, then the topology with the highest log likelihood value was chosen. The branch lengths were calculated by the number of residue substitutions per site, and the tree was depicted to scale. MEGA7 software was used to display the evolutionary analysis (Kumar et al., 2016).

## Results

### Real-Time RT-PCR (rRT-PCR)

Based on rRT-PCR molecular diagnosis, the four cat samples and the veterinarian sample were SARS-CoV-2 positive (as illustrated in **Supplementary Table S3**) with cycle threshold (CT) values of nucleocapsid (N) and ORF1ab genes.

### Sequencing and Phylogenetics

**Figure 3:**
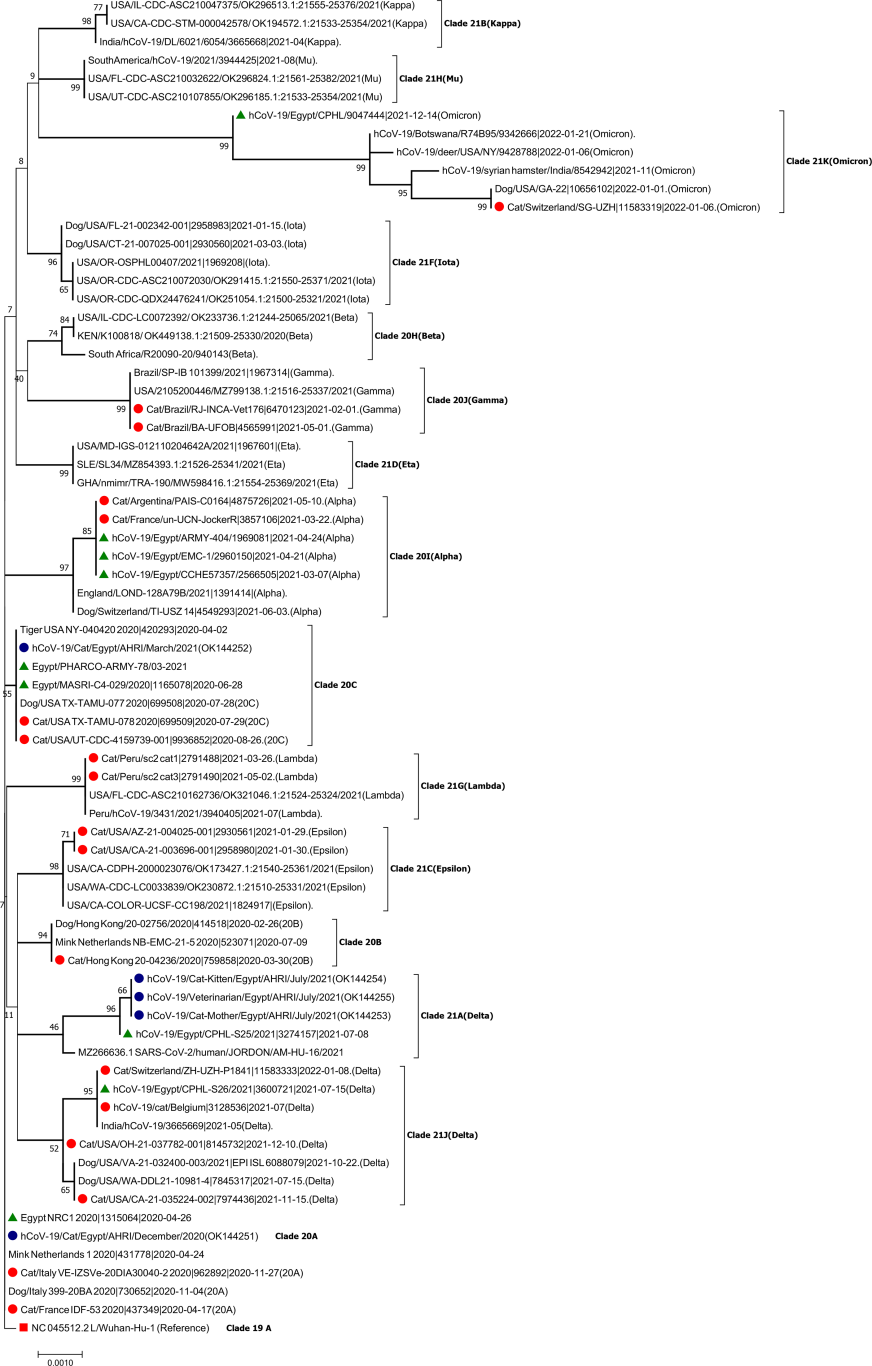
Phylogentic analysis of the SARS-CoV-2 sequences. The rooted maximum likelihood tree shows the relationship between sequenced samples and current circulating variants in humans and animals in which the related taxa and closed branches are clustered together illustrating SARS-CoV-2 evolution. The blue circles illustrate the sequenced SARS-CoV-2 that were detected in cats in the present study while the red circles display the detected SARS-CoV-2 in cats worldwide from 2020 till January 2022. The green triangles depict circulating SARS-CoV-2 strains in Egypt within the same time frame. The red square refers to SARS-CoV-2 Wuhan-Hu-1 human reference strain.

All obtained sequences of the five samples were uploaded to the National Centre for Biotechnology Information (NCBI) with accession numbers OK144251 to OK144255. Full spike gene sequencing and phylogenetic analysis revealed that sample hCoV-19/Cat/Egypt/AHRI/December/2020(OK144251) belonged to clade 20A and illustrated high identity with NC 045512.2 L/Wuhan-Hu-1 (reference strain) and Egypt_NRC1_2020| EPI_ISL1315064|2020-04-26 (99.9%–100%). hCoV-19/Cat/Egypt/AHRI/March/2021(OK144252) was quite identical to Egypt/PHARCO-ARMY| EPI_ISL1936365/03-2021 with high identity (99.9%), and both of them are related to clade 20C. On the other hand, hCoV-19/Cat-Mother/Egypt/AHRI/July/2021(OK144253), hCoV-19/Cat-Kitten/Egypt/AHRI/July/2021(OK144254) and hCoV 19/Veterinarian/Egypt/AHRI/July/2021(OK144255) were similar to a human sample collected in the same month (hCoV-19/Egypt/CPHL-S25/2021| EPI_ISL3274157|2021-07-08) with high identity (99.7%), and they belonged to clade 21A (Delta). The latter samples had some similarity with MZ266636.1 SARS-CoV-2/human/JORDON/AM-HU-16/2021 with high identity (99.5%), as presented in **Figure 3** and **Supplementary Table S4**.

### Amino Acid Substitution

In this study, seven critical amino acid substitutions were observed in spike glycoproteins, all of them were non-synonymous, namely (W152R; S:T454A) in the N-terminal domain of the S1 subunit, (L452R; S:T1355G) in the receptor-binding motif **(**illustrated in **Supplementary Figures S2B, C**), (A570V; S:C1709T) in C-terminal domain 1 (CTD1), (D614G; S:A1841G) which is found downstream of the receptor-binding domain in C-terminal domains 2 (CTD2), (Q677H; S:G2031T) in the S1/S2 cleavage site, (A899S; S:G2695T) in the fusion-peptide proximal region (FPPR) and (S1051Y; S:C3152A) in the β-hairpin region of subunit 2 (S2). Interestingly, some of these mutations have not been previously detected in cat samples in Hong Kong, France, Italy or the USA. Hence, this is the first report of these mutations in cats. The latter mutations were (W152R), (A570V), (Q677H), (A899S) and (S1051Y), as shown in **Table 1**.

**Table 1:** Amino acid substitutions in the sequenced samples in comparision to some circulating variants in human and cats.

No		Region/Amino acid Position						
		NTD	RBM	CTD1	CTD2	S1/S2	FPPR	β-hairpin
		W152	L452	A570	D614	Q677	A899	S1051
**0**	NC 045512.2 L/Wuhan-Hu-1 (Reference)	**W**	**L**	**A**	**D**	**Q**	**A**	**S**
**1**	Cat Hong Kong 20-04236/2020|759858|2020-03-30	.	.	.	G	.	.	.
**2**	Cat_France_IDF-53_2020|437349|2020-04-17	.	.	.	G	.	.	.
**3**	Cat_France_Env-Ba_2020|483063	.	.	.	G	.	.	.
**4**	Cat_Italy_VE-IZSVe-20DIA300402-2020|962892|2020-11-27	.	.	.	G	.	.	.
**5**	Cat_USA_TX-TAMU-078_2020|699509|2020-07-29	.	.	.	G	.	.	.
**6**	hCoV-19/cat/Belgium|3128536|2021-07(Delta)(4.1)	.	R	.	G	.	.	.
**7**	hCoV-19/Cat/Egypt/AHRI/December/2020 (OK144251)*	.	.	.	G	.	.	.
**8**	Egypt_NRC1_2020| EPI_ISL1315064|2020-04-26	.	.	.	G	.	.	.
**9**	hCoV-19/Cat/Egypt/AHRI/March/2021 (OK144252)*	.	R	.	G	H	.	.
**10**	Egypt/PHARCO-ARMY|EPI_ISL1936365/03-2021	.	R	.	G	H	.	.
**11**	hCoV-19/Cat-Mother/Egypt/AHRI/July/2021 (OK144253)*	R	R	V	G	H	S	Y
**12**	hCoV-19/Cat-Kitten/Egypt/AHRI/July/2021 (OK144254)*	R	R	V	G	H	S	Y
**13**	hCoV-19/Veterinarian/Egypt/AHRI/July/2021 (OK144255)*	R	R	V	G	H	S	Y
**14**	hCoV-19/Egypt/CPHL-S25/2021| EPI_ISL3274157|2021-07-08	R	R	.	G	H	S	.
**15**	MZ266636.1 SARS-CoV-2/human/JORDON/AM-HU-16/2021	R	R	.	G	H	S	.

*The sequenced samples in this study.

### The Overall Distribution of Mutations and Impact on the Structure and Stability of the Spike Protein

**Figure 4:**
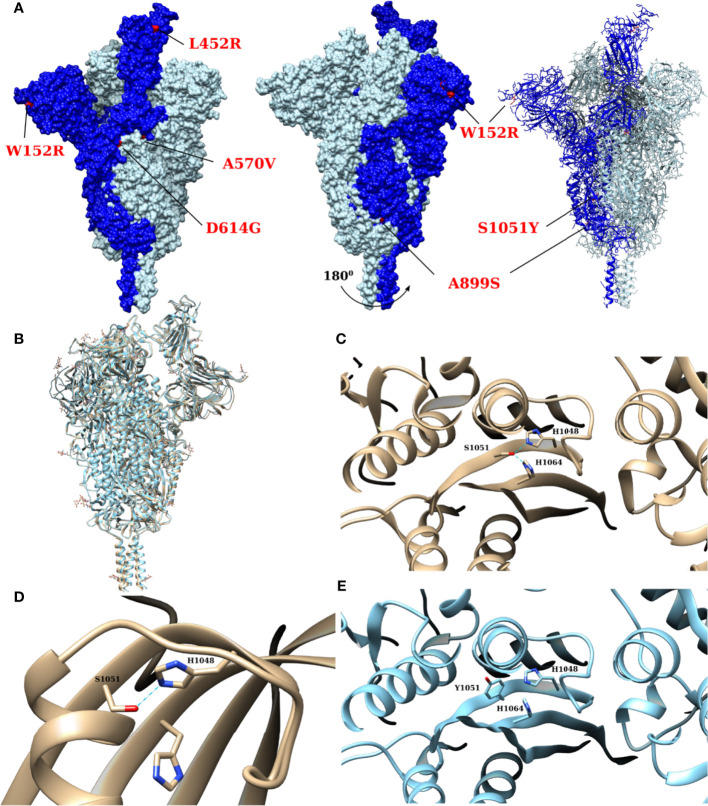
The overall distribution of the spike protein mutations observed in the studied sequences and the impact of (S1051Y) mutation on its structure and function. **(A)** shows the overall distribution of the mutations, found in the sequenced samples in this study, mapped onto the predicted structure by homology modelling. The mutated residues in one monomer are depicted in red and the monomer with RBD-up is depicted in dark blue. The isosurface of the trimer is shown on the left and a rotated view (by 180◦) of the latter is shown in the center, while the atomic details are shown on the right. **(B)** illustrates the predicted structure of the sequence presented in this study, depicted in light blue, superposed onto the template used to generate it (depicted in gold, derived from PDB ID 7krr). **(C–E)** illustrate molecular details of the impact of (S1051Y) on the structural stability and function of spike protein. In the presence of the native serine 1051 (PDB ID 7krr), the residue interacts with two histidine residues (His 1048 and His 1064), as illustrated in **(C)** (top view, showing hydrogen bonds depicted by the cyan lines) (Zhang J et al., 2021). Such interactions are likely to stabilize the beta-sheet within the native conformer. These interactions are likely to be abolished by the mutation (S1051Y), as shown in the predicted model by homology modelling **(E)**. **(D)** shows the interaction of the serine and the two histidine residues in the unmutated post-cleavage structure [PDB ID 6xra (Cai et al., 2020)]. The figure illustrates that post-cleavage structural changes in the protein lead to bond break between the serine residue and His 1064.

The overall distribution of the mutations mapped onto the predicted structure (homology model) of the studied sequence (**Figure 4**) showed that four residue substitutions were located on the viral surface (**Figure 4A**), which were (W152R), (L452R), (A570V) and (A899S). Furthermore, the (D614G) mutation, which is found downstream of the receptor-binding domain (RBD), leads to a loop misplacement that results in structural rearrangements (Zhang J et al., 2021).

More specifically, in the closed trimer state, the D614 structure was found to have disordered loops between residues 620 and 640 (designated as 630 loops) within its monomers. Upon mutation, the G614 showed ordered loops instead, as a consequence of the elimination of a salt bridge between D614 of CTD2 and K854 in the FPPR. It was concluded that such a mutation leads to the stabilization of the closed trimer state. On the contrary, the one RBD-up state requires at least one of the loops to be disordered and the second one to be partially disordered. The latter state precedes the formation of two and three RNA-up states, which are the precursors of the S2 post-cleavage state. The (D614G) mutation, therefore, slows down the formation of one RBD-up state, as well as the shedding of the S1 before the post-cleavage state formation. The accumulation of the one RBD-up state, but not the other RBD-up states, is thought to explain the increased infectivity, but at the same time, the reduced overall binding affinity with the receptor. As a consequence, the formation propensity of a post-cleavage state is decreased in viral strains with a (D614G) mutation (Zhang J et al., 2021).

The (A570V) and the (S1051Y) mutations are of particular interest as they are located at the interface of different chains and domains. **Supplementary Figure S1** illustrates that the residue 570 is surrounded by several other hydrophobic residues in both native and predicted structures. The increase in hydrophobicity of V570 compared with A570 suggests that this mutation is likely to contribute to the stabilization of this residue in place.

The impact of (S1051Y), which is located in the β-hairpin region of subunit 2 (S2), is illustrated in **Figures 4C–E**. The pre-cleavage structures of the native sequence, in both the closed and open conformations, show that the residue S1051 is in contact with two histidine residues, namely H1048 and H1064, as illustrated in **Figures 4C–E**. In the closed pre-cleavage structure, the distances between the hydroxyl oxygen of the serine S1051 to the nearest nitrogen atoms on the rings of H1048 and H1064 are 2.71 and 2.77 Å, respectively. One of the two histidines, H1048, is on the same side of the β-sheet strand as S1051, whereas the other (H1064) is on the other strand of the β-sheet hairpin. The serine residue is therefore bridging the interaction between the two histidines, effectively stabilizing the hairpin and the overall structure of the β-sheet. In contrast, the post-cleavage structure shows that one of the two histidines (H1064) moves away from the serine residue, whereas 1048 remains in close proximity. The distances between the serine oxygen and the closest ring nitrogen atoms of the histidines are 2.82 and 4.4 Å for H1048 and H1064, respectively. This is due to a shift that is seen of the relative orientation of the two strands of the β-hairpin upon the unfolding of the helices in the post-cleavage conformation. Such large conformational changes increase the mechanical stress on the β-hairpin structure as well as on the nearby disulphide bridge. The predicted model of the (S1051Y) mutation shows that the histidine interactions are diminished in the pre-cleaved conformer, which may contribute to the destabilization of the hairpin and the overall stability of the β-sheet. On the other hand, these hydrogen bonds break may contribute to lowering the energetic barrier to the large conformational changes required for the post-cleavage structure to be formed.

### Structural Modeling of the ACE2 Receptor in Various Species and Their Phylogenetic Analysis Deciphers Host Susceptibility

As the mutations that were found in the cat samples have been previously reported in humans, there must be other factors that determine the host range of the virus, including viral entry into the host cell. To further understand the factors that control host range and host susceptibility, we compared the sequences of the host receptor, ACE2 (angiotensin-converting enzyme 2), in different hosts. The ACE2 receptor is a highly conserved protein that performs a specific enzymatic function in mammals. We performed an initial alignment of the ACE2 protein isoform 1 from humans with that of susceptible companion animal hosts, (namely, cats and dogs) and with a species that have not been reported to be infected with SARS-CoV-2 (i.e., an unsusceptible control species). For the latter purpose, we used the ACE2 protein from chickens.

In this initial comparison, we focused on the residues that have been shown to have direct contact with the virus RBD domain (e.g., from PDB IDs 6LZG and 6M17). **Table 2** shows a comparison between the key residues that form such polar and hydrophobic contacts. One can ascertain that the number of residues substituted in such a way that alters the charge distribution of the binding interface in dogs (3 out of the 10 residues are substituted) is more than that in cats (2 out of the 10 residues are substituted), and that such substitutions are the most in chickens (7 out of the 10 residues are substituted). We also noticed that there seems to be no clear pattern or preference in these substitutions, but rather an overall change in the charge distribution was observed. More specifically, a polar to hydrophobic residue substitution Q24L is seen in cats and dogs. Whereas in chickens, a polar to a charged residue substitution Q24E is seen. Residues D31 in humans or E31 in cats and dogs, are substituted with A31 in chickens. The polar and positively charged residues Q32 and K31, respectively, which are conserved in humans, cats and dogs are substituted with negatively charged residues (K31E and Q32E) in chickens. The hydrophobic methionine residue M82, is substituted with a polar residue in cats and dogs, and even with a charged residue in chicken. Finally, the tyrosine residue Y83, which is conserved in human cats and dogs, is substituted with a more hydrophobic residue (phenylalanine) in chickens. Additionally, the alignment showed several residues on the surface of the receptor in close proximity to those that form direct contacts with the RBD, which undergo substitutions that also alter the binding interface charge distribution in unsusceptible species compared with susceptible ones. It is possible that these neighboring residues may affect the overall binding affinity of the viral RBD with the host receptor. For instance, some substitutions may introduce an overall negative charge (e.g., the substitution of E35 in humans, cats and dogs, to R35 in chickens), increase hydrophobicity or even introduce stearic barriers. These observations suggest that the overall residue composition and associated charge distribution on the binding interface may be taken as an indicator (or at least a contributing factor) of the susceptibility of the host. The latter also suggests that the homology of the 10 sites on the ACE2 that make direct contact with the RBD, between humans and other hosts, may not alone be an efficient indicator of the host susceptibility, and a better indicator would be to compare all residues that are at or near the binding interface.

**Table 2:** Substitutions in key residues involved in viral recognition on the ACE2 surface in selected species.

Human	Cat	Dog	Chicken
Q24	L	L	E
D30	E	E	A
K31	K	K	E
H34	H	Y	V
Y41	Y	Y	Y
Q42	Q	Q	E
M82	T	T	R
Y83	Y	Y	F
K353	K	K	K
R357	R	R	R

The table shows key residues involved in the binding of the human ACE2 with the viral RBD. The structure showed key residues that form a set of polar contacts (as well as one hydrophobic contact) with the viral RDB, hence stabilizing the complex. Substitutions that increase the hydrophobicity and polarity of the binding surface with respect to the human receptor are depicted in gray and orange fonts, respectively. Whereas substitutions that increase negative and positive charge are depicted in red and blue fonts, respectively.

**Figure 5:**
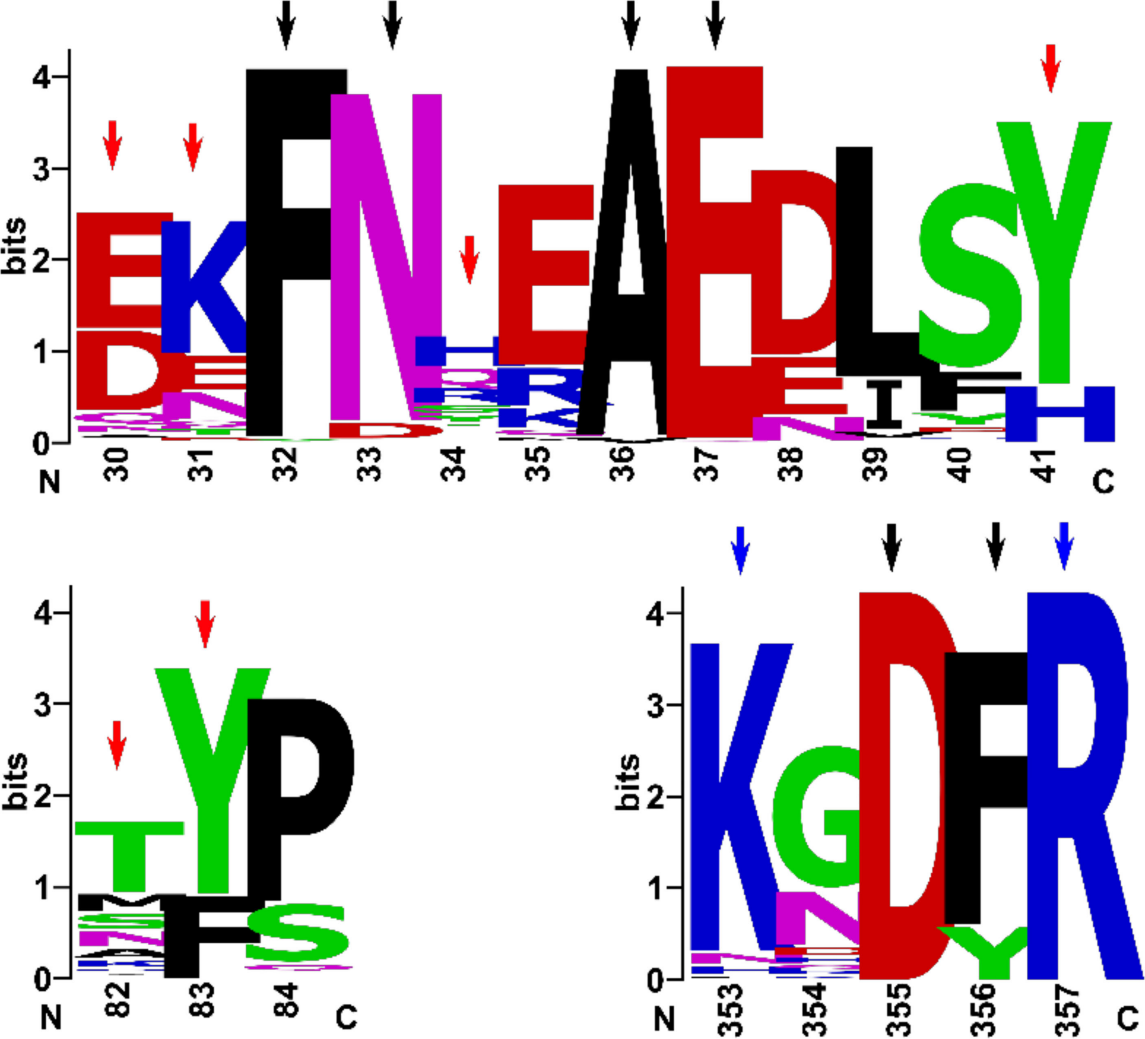
Conservation of residues involved in direct contact between the receptor and the viral RBD. The figure shows sequence logo representation of alignments of residues 30-41, residues 82-84 and residues 353-357 of the host receptor (ACE2) from different species. The alignments involved 249 sequences that had the highest identity with the human ACE2 isoform 1 sequence. Highly conserved residues in this region are marked with black arrows. Conserved and non-conserved residues that are involved in direct contact with the viral RBD are marked with blue and red arrows, respectively.

To study this on a wider range of animal species, we performed a BLASTP search against the protein reference sequence database and retrieved sequences from different animal species with the highest sequence identity compared with the human ACE2 peptidase domain sequence (see methods for details). The multiple sequence alignment of the latter containing 249 sequences was used for further analysis. Indeed, sequence logo representations (**Figure 5**) of the alignment show that certain residues of those that form direct contact with the viral RBD, along with neighboring residues, are conserved among many animal species. More specifically, residues F32, N33, A36, E37, D355, F356, K353 and R357 (marked with black and blue arrows in **Figure 5**) are highly conserved in the analysed sequences of the different animal species and are present at the receptor-RBD binding interface. Residues that are in direct contact with the viral RBD (mostly through their side chains) are marked with blue or red arrows. K353 and R357 are highly conserved and are in direct contact with the viral RBD. Whereas, residues D30, K31, H34, Y41, M82 and Y83 (marked with red arrows), along with two other residues (Q24 and Q42), are also directly involved in the binding, but show moderate to high variations among the different animal species. Hence the extent of substitutions in the latter (variable) residues can provide a preliminary indication of how similar is the binding capacity of the different animal receptors compared to human. Neighbouring highly variable residues at the binding interface may, however, such as E35, D38 and G354 although are not in direct contact with the viral RBD in the human receptor, may have substitutions that would enhance or interfere with binding in other species. These findings confirm the previous notion that considered that all residues at or near the binding interface would form a better indicator for the likelihood of viral RBD binding with a specific host.

**Figure 6:**
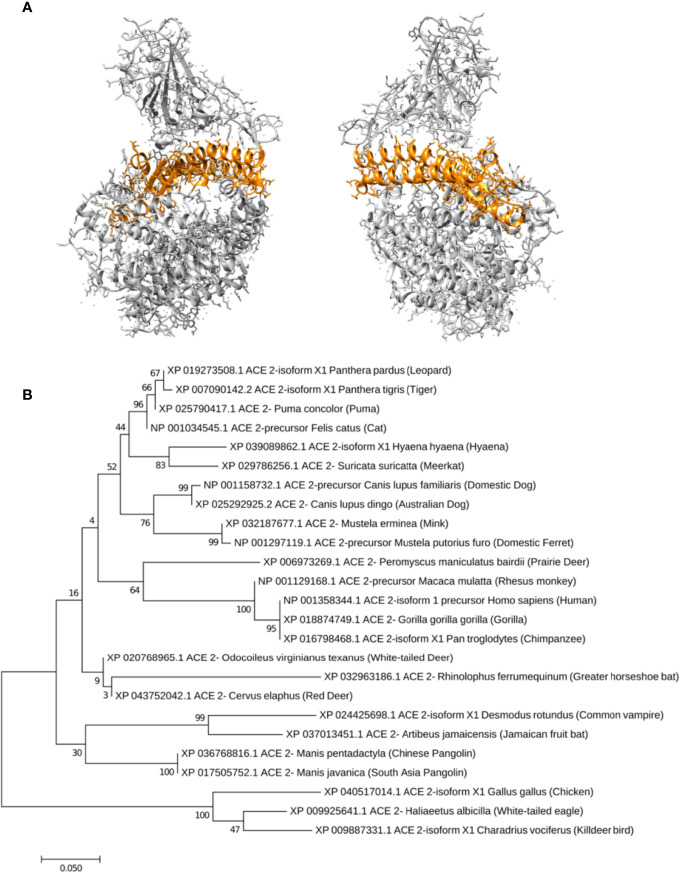
Phylogenetic analysis of the residues near the receptor-viral RBD interface. **(A)** shows the structure of the human ACE2-viral RBD complex (PDB ID 6lzg), for both front and 180 degrees rotated views, with the residues at or near the receptor-viral RBD depicted in orange, whereas the remainder of the ACE2 receptor PD domain and the viral RBD domain are colored in light grey. The alignment of latter residues (19-90 and 324-393) were used to construct a phylogenetic tree, shown in **(B)**. The latter tree in panel B demonestrats a midpoint rooted maximum likelihood where the related taxa and closed branches are clustered together. The tree displays the evolutionary analysis of ACE2 receptor’s residues in contact with the viral RBD in different species and their relationship.

Based on the latter arguments, and for the purpose of finding a simple but quantitative indicator of virus entry and susceptibility based on comparative sequence analysis with the human sequence, we constructed a phylogenetic tree (**Figure 6**) using the alignment blocks of all the residues at the binding interface between the ACE2 and the viral RBD. Residues 19–90 and 324–393 (shown in orange color in **Figure 6A**) were selected for this purpose.

The phylogenetic analysis and identity matrix of all the aligned residues at the binding interface between the ACE2 and the viral RBD revealed that this region in humans, gorillas and chimpanzees are closely related to each other and are identical (100%). The latter region identity between humans and cats (83.8%) is higher than that of humans and dogs (79.5%). While the identity is 78.1% in ermines (minks) and 77.4% in ferrets, it is quite low in chickens (64.7%), as presented in **Figure 6B** and **Supplementary Table S5**.

**Figure 7:**
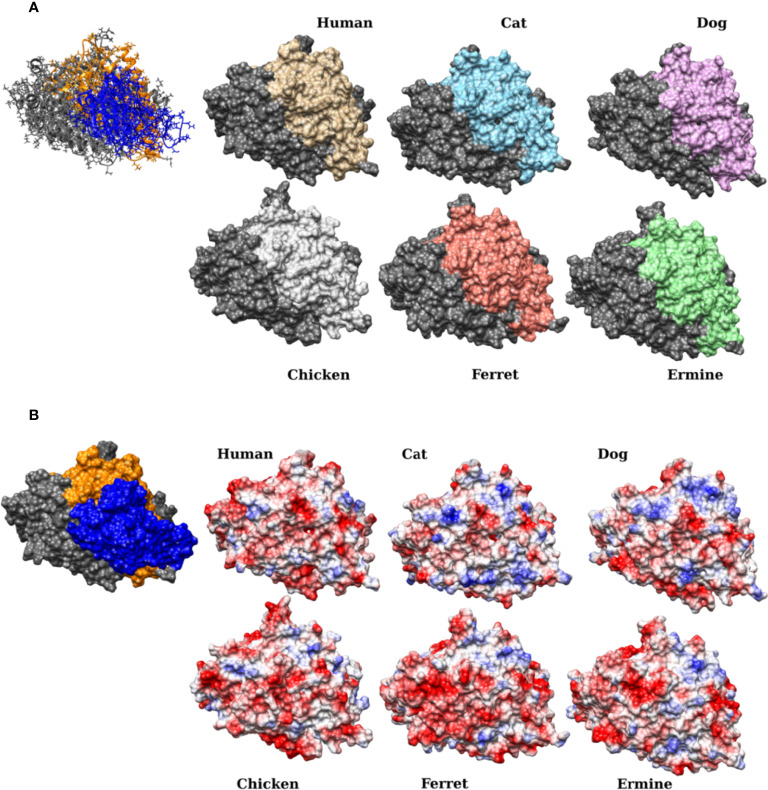
Isosurface representations and electrostatic (Coulombic) maps of the predicted ACE2 receptor structures for various animal hosts. The figure shows the isosurface representations of the predicted structures of the ACE2 **(A)** and the corresponding Coulombic coloring of the surface representations **(B)**. The region that is likely to affect the binding (residues 19-90 and 324-393) is depicted in distinct colors whereas the rest of the PD domain is depicted in dim grey. The viral receptor is hidden to show the overall shape and surface charge of the binding surface on the ACE2 receptor. For clarity, the human receptor-RBD complex is shown on the left of both atomic and its isosurface representations (panels **A** and **B**, respectively), in the same orientation as of the other models. In the latter, the RBD is in blue, the region having residues 19-90 and 324-393 is depicted in orange and the remainder of the PD domain is in dim grey. In panel B, the positively charged, negatively charged, and hydrophobic (neutral) regions are depicted in blue, red and white, respectively.

To visualize and investigate the impact of mutations at the binding interface, homology models were built for the receptor-RBD complex, by fixing the sequence of the viral RBD and varying the receptor sequence according to the species (**Figure 7**). Models were built using the PDB IDs 6LZG (Wang et al., 2020) and 6M17 (Yan et al., 2020) as templates. **Figure 7A** shows the isosurface representations of the obtained models (**Supplementary Figure S2**) for the receptor PD of the selected species, namely human, cat, dog, ferret, ermine and chicken. The binding interface is colored in different colors, whereas the remaining part of the receptor domain is colored in dim gray. An analysis of these models revealed that, on the one hand, the overall shape and structure of the binding interface region of the cat and the dog are more similar to that of the human when compared with their counter regions in the ferret and the chicken. These larger variations in the latter (less susceptible) species may introduce stearic effects that may reduce or hinder the binding. On the other hand, the electrostatic (Coulombic) colored isosurfaces (**Figure 7B**) show the appearance of several hydrophobic regions within the binding region (shown in white), as well as an increase in the positive charges (shown in blue), in the cat and the dog compared with that of the human receptor. The latter positively charged regions appear to be more in the dog than in the cat. In contrast, the surface of the ferret and the chicken receptors appear to be more negatively charged. Similar conclusions can be drawn on the analysis of polar contacts and potential hydrogen bonding in the same models, i.e., between the RBD and receptors from the different species (**Supplementary Figure S2**). It is clear that the number of polar contacts in cats and dogs are less than in humans. Out of the studied species, the chicken has the least contacts. The ermine and the ferret had a different distribution of predicted hydrogen bonds when compared with humans.

## Discussion

Coronaviruses infect a wide variety of hosts (Shafique et al., 2020). In the present study, we aimed to investigate the mutations of the SARS-CoV-2 spike glycoprotein detected in cats and their effect on its structure and function by sequencing the full spike gene, followed by bioinformatics analysis. Furthermore, we highlighted the effect of residues substitution on viral infectivity, evolution and evasion from immune response. We found that cats with a history of direct contact with COVID-19-positive owners were also infected with COVID-19, revealing the possibility of SARS-CoV-2 transmission between humans and cats.

Generally, the high substitutions and mutations in coronaviruses revealed that the virus is adapting to new hosts (Duffy, 2018). The significance of amino acid substitution depends on the switching of properties and classes; for instance, a spike glycoprotein switching from leucine to methionine in L452M may have no significance as both are hydrophobic and neutral. Similarly, switching from tyrosine to phenylalanine in the Y453F mutation may have no significance as both are aromatic and hydrophobic. In contrast, substitution from glutamine to histidine in the Q498H mutation could significantly change the protein and side chain structure because they have different features, as histidine has a positive charge while glutamine has a neutral charge (Elaswad et al., 2020). Therefore, significant changes in a spike glycoprotein could change its viral infectivity, pathogenicity and virulence, and could increase cross-species transmission (Wan et al., 2020).

In the present study, SARS-CoV-2 incidence in cats was detected from four clinical samples, and was consistent with molecular results reported in previous studies (Newman et al., 2020). Furthermore, previous experimental studies have shown the clinical susceptibility of cats to SARS-CoV-2 (Hamer et al., 2021), which may be due to the similarity in ACE2 receptors between feline species and humans (Sailleau et al., 2020; Patterson et al., 2020; Hamer et al., 2021; Ruiz-Arrondo et al., 2021). Moreover, cats shed SARS-CoV-2 horizontally after infection for up to 6 days (Hamer et al., 2021), and the virus can persist in cats post-infection for up to 32 days (Bosco-Lauth et al., 2020; Shi et al., 2020). The high positive incidence in our study can be attributable to the fact that all tested cat samples came from homes with COVID-19-positive human cases.

SARS-CoV-2 transmission between humans and cats was reported in this study as the results of the phylogenetic analysis revealed a similarity between SARS-CoV-2 detected in cats (OK144251, OK144252 and OK144253 to OK144255) and in human cases reported in the same months (EPI_ISL1315064, EPI_ISL1936365 and EPI_ISL3274157), as presented in **Figure 3** and **Supplementary Table S4**. Additionally, the veterinarian who was in contact with the infected cats was asymptomatic; however, he tested positive for COVID-19 and his sequence result was identical to that of the young cat and her mother. Moreover, sequence similarity between the SARS-CoV-2 spike gene detected in the young cat and her mother confirms that the virus is the same. Thus, there is a potential support of SARS-CoV-2 transmission between humans and cats, but further studies are needed to investigate the direction of the viral transmission as the study’s design has not determined the infection direction whether animal-to-human or human-to-animal or animal-to-animal viral infection occurred.

The present study revealed changes in the spike glycoproteins and the unique amino acid substitutions of the cat samples in correspondence with the reference Wuhan isolate, as illustrated in **Table 1**, **Figure 4** and **Supplementary Figure S1**. Seven critical amino acid substitutions were observed. The diverse and frequent single residue mutations that emerged independently and repeatedly suggests their possible adaptive role (Kubik et al., 2021). Different spike regions are subjected to distinct evolutionary changes as mutations at receptor-binding domains (RBDs) result in both changes in the binding affinity to ACE2 receptors as well as in viral evasion from neutralizing antibodies, whereas mutations in N-terminal domains (NTDs) are mostly related to the potential escape from neutralizing antibodies (Kubik et al., 2021). Interestingly, some of these mutations are unique to the Wuhan-Hu-1 reference isolate and are reported in cat samples for the first time in this study, as they were not previously detected in cat samples in Hong Kong, France, Italy or the USA. The latter mutations are (W152R), (A570V), (Q677H), (A899S) and (S1051Y). In this study, we focused on the spike gene as it the most variable part of the viral genome and it plays crucial role in the receptor binding. It is also the protein that carries the receptor binding domain (RBD) which is the main point of entry to the host (Fung and Liu, 2019). The spike is also the main protein that exhibit mutations due to immune escape and generally have a major immunological role (Harvey et al., 2021).

Structural visualization and homology models revealed that four residue substitutions were located on the viral surface, which may have appeared as viral antigenic evolution to evade neutralizing antibodies. These mutations, (W152R), (L452R), (A570V) and (A899S), have weakened critical interaction points with the neutralizing antibodies and are associated with low sensitivity to mAbs and vaccine sera (Kubik et al., 2021). The observed (W152R) mutation is critical and is frequently observed across geographical and phylogenetic contexts; thus, it is a hotspot for mutations (Kubik et al., 2021). The substitution of tryptophan to leucine (W152L) or arginine (W152R) is the most common mutation at this location based on the SOPHiA DDM database. In addition, the substitution of tryptophan to cysteine (W152C) or glycine (W152G) is rare (Sophiagenetics, 2021). During the second wave of the SARS-CoV-2 pandemic, between December 2020 and April 2021, the (W152R) mutation increased steadily and rapidly, with one of the most notable adaptive mutations (N501Y) in the receptor-binding domain (RBD) coexisting (Kubik et al., 2021). Similarly, (W152R)substitution affects viral evasion from neutralizing antibodies and escape from immunity, as demonstrated in structural studies in which tryptophan (W) removal has weakened critical interaction points with antibodies (Kubik et al., 2021). In particular, a residue in the antibody chain interacts with W152 in a hydrophobic pi-stacking interaction, wrapping W152 inside one of the complementarity-determining region (CDR) loops in the variable chains of antibodies (Kubik et al., 2021). Indeed, W152 is a key position that contributes to the latter interaction. As a result, changes in W152 impair not only the pi-stacking interaction, but also the pocket created by the antibody’s CDR loop, resulting in a significant reduction in binding affinity (Kubik et al., 2021). Furthermore, (W152R) substitution suggests viral evolution to maintain effective binding to the ACE2 receptor (Kubik et al., 2021). Therefore, monitoring the individual mutations that occur outside the receptor-binding domain (RBD) and inside the N-terminal domain (NTD) is critical.

The second mutation observed in this study was (L452R) in the receptor-binding motif (RBM), as illustrated in **Supplementary Figures S2B, C**. This mutation was first reported in January 2021 in California, USA in the 20C/S:452R variant, and its frequency has increased over time (Zhang W et al., 2021). Since the first observation, novel variants of SARS-CoV-2, including this amino acid substitution, have been seen in the Delta/21A/B.1.617.2, Kappa/21B/B.1.617.1 and Epsilon/21C/B.1.427.9 viruses with increased virulence and infectivity (Lauring and Hodcroft, 2021). Additionally, in this mutation, a factor of a 3- to 6-fold reduction in antibody neutralization titers in vaccinated individuals was observed (McCallum et al., 2021). Furthermore, this substitution reduced the neutralizing activity of monoclonal antibodies against RBD by 14- to 35-fold (McCallum et al., 2021). Moreover, a 2-fold increase in viral shedding was reported in the involved variants (Deng et al., 2021). It was also found that (L452R) could strengthen ACE2 receptor-binding affinity (Chen et al., 2020) and increase viral infectivity (Liu et al., 2021). A mutation in the C-terminal domain 1 (CTD1) at position 570 was also observed. In addition, (A570V) was detected in North America and Asia (Guruprasad, 2021). Recently, biophysical and Cryo-EM studies demonstrated that some residue substitutions in the spike have affected the conformational dynamic equilibrium between the open RBD-up and closed RBD-down forms of the spike (Henderson et al., 2020). In particular, the authors found that certain residue substitutions in the regions of contact between domains could affect the equilibrium between the open and closed states. For example, the combined mutations in the A570, T572, F855 and N856 positions of SD1 and S2 inter-protomer regions have led to the dominance of the open RBD-up receptor-accessible form of the spike. Therefore, these structural rearrangements have resulted in the enhancement of the binding affinity of the spike to the ACE2 host receptor (Henderson et al., 2020). More specifically, the substitution of A570 with a more hydrophobic one, such as L570, has aided in such an equilibrium shift. Hence, the hydrophobic (A570V) mutation (**Supplementary Figure S1**) observed in our samples may contribute to a similar equilibrium shift. The fourth mutation on the surface was (A899S) in the fusion-peptide proximal region (FPPR). This mutation was recently observed in Jordan (Alanagreh et al., 2021), Saudi Arabia (Alghoribi et al., 2021) and Italy (Castelli et al., 2021). This substitution is recorded in lineage C.36 and is associated with high transmissibility and low sensitivity to mAbs and vaccine sera (Castelli et al., 2021).

The fifth mutation in our study (D614G) was found downstream receptor-binding domain in the C-terminal domains 2 (CTD2). Such a mutation became dominant during the emergence of the COVID-19 pandemic (Korber et al., 2020a; Yurkovetskiy et al., 2020). It is not only able to enhance the viral/host interaction on the cellular level (Ortega et al., 2020), but it is also involved in the upward increase in SARS-CoV-2 pathogenicity, increasing viral transmission and evasion from immune response and vaccine (Korber et al., 2020b). This mutation appears to enhance the spread of SARS-CoV-2 and increases the viral infectivity that resulted from increasing the stability of the spike trimer. Certainly, in such a mutation, there is loop misplacement, structural rearrangements and interaction between downstream receptor-binding domains, which act as wedges within the spike trimer that strengthen the stability and prevent pre-maturation of the spike, and improve the strong assembly of the virus, resulting in the enhancement of the viral binding with the ACE2 receptor, as presented in **Figure 4**. This could facilitate viral entry, infectivity and virulence. It is unsurprising that all newly emerged variants contain this mutation and that they are more infectious than the Wuhan reference virus (Zhang J et al., 2021). The (D614G) substitution was reported to improve viral replication in human lung epithelial cells and primary human airway tissues by improving viral infectivity and stability (Plante et al., 2021). Furthermore, this mutation lowered the effectiveness of COVID-19 vaccinations in clinical studies (Plante et al., 2021).

The (Q677H) mutation in the S1/S2 cleavage site was detected in both the two cat samples and the veterinarian sample in this study. The (Q677H) mutation was firstly identified in clade 20C in the USA (Pater et al., 2021). It was also detected in the (Eta/21D/B.1.525) variant (Hodcroft et al., 2021). As the position of such a mutation is found in a critical polybasic region of the furin-binding site and it could influence S1/S2 cleavage, SARS-CoV-2 infectivity and pathogenicity could be subsequently affected (Hodcroft et al., 2021). The seventh mutation observed in this study is (S1051Y) in the β-hairpin region of subunit 2 (S2). The three-dimensional organization of HR1 (residues 910−985), CH regions (residues 986−1035), β-hairpin (1035–1068) and UH (residues 736−781) is a significant feature of the spike glycoprotein structure (Walls et al., 2016). Structural modeling and analysis suggest that the (S1051Y) mutation (**Figures 4C–E**) structure. Indeed, this mutation could play a role in viral entry during the replication cycle. The latter hypothesis requires further experimental investigation.

Despite the multiple mutations found in the sequences presented in this study, all these mutations had been previously reported in humans. Hence, although some of the latter mutations may be important for the virus strain survival within cats, these mutations are not unique to this particular host, and it is likely that a cross-infection between humans and cats has occurred. These findings raise several questions, including: What factors control the SARS-CoV-2 host ranges? Do variations in the host receptor play a role in the susceptibility of the host to SARS-CoV-2 infection? Can we obtain insights into the susceptibility of a species by comparing the species receptor with the human receptor? To answer these questions, we analyzed the ACE2 receptors from various animal species using sequence and structural bioinformatics tools.

Obviously, many host factors, may play a crucial role in susceptibility to SARS-CoV-2 infection. One of the possible factors likely to influence the virus host range is the variation in the viral receptor itself from one species to another. In this work, we showed that simple sequence comparisons (**Table 2** and **Figure 5**), phylogenetic analysis (**Figure 6**) and the structural modeling (**Figure 7**) of residues at the binding interface between ACE2 receptors from different hosts and the viral RBD may be used as an initial effective indicator of the susceptibility of the host to SARS-CoV-2 cross-infection. More specifically, the amino acid composition of this region (residues 19–90 and 324–393) may provide an indication of the binding capacity of the virus to the specific species host cell, and hence a determinant for the susceptibility of the host. This study, therefore, reveals that a simple phylogenetic analysis and identity matrix of the aligned residues could provide a quantitative measure of the likelihood of virus entry into the cells of various hosts.

The phylogenetic analysis and identity matrix of the aligned residues at the binding interface between the ACE2 and the viral RBD (**Figure 6B** and **Supplementary Table S5**) revealed that SARS-CoV-2 has the ability to interact with a broad range of ACE2 cell receptors in different species, which could increase its virulence and could enhance its inter-species transmission (Su et al., 2016) (Bolles et al., 2011). The sequence identity of the latter region between humans and cats (83.8%) is higher than that between humans and dogs (79.5%), which suggests that the potential recognition of the RBD-spike glycoprotein in cat ACE receptors is stronger than that in dogs, and these results are in accordance with a previous study in China (Luan et al., 2020). This high sequence identity in cats compared to human could explain the relatively high SARS-CoV-2 infection rate in the feline species (Hamer et al., 2021). Hence, regular monitoring of companion animals for SARS-CoV-2 infection is highly recommended. The similarity percentage in ermines (minks) (78.1%) is lower than that in cats and dogs, however the reported infection rate of SARS-CoV-2 in minks is high, according to the fifth report of the OIE (2021). This may be an indicator that there are other effective factors in minks which make this species more susceptible to SARS-CoV-2 than others, and these factors need further investigation. The sequence identity was 74.6% in greater horseshoe bats and 78.8% in Chinese pangolins. Previous studies have suggested that SARS-CoV-2 from bat CoV RaTG13 and pangolin isolates, have sequence identities of 96.3% and 89%, respectively. These studies concluded that, bats could be the natural origin of SARS-CoV-2 (Bosco-Lauth et al., 2020; Shi et al., 2020), and although pangolins cannot transmit SARS-CoV-2 directly due to the percentage of divergence, they may play an intermediate role (Calvet et al., 2021). Indeed, there is a need for further investigation to discover other factors that may point to bats being the potential natural host for SARS-CoV-2, rather than the receptor homology and binding affinity. Overall, the presented work paves the way for binding affinity predictions and free energy calculations through molecular dynamics studies, and opens the gates for further investigation into species susceptibility to SARS-CoV-2 infection.

## Conclusions

This study displays critical mutations in the spike glycoprotein of SARS-CoV-2 detected in cats and in their treating veterinarian, through full spike gene sequencing, structural visualization and homology modeling. The analysis of the detected mutations revealed potential evidence for spillover of SARS-CoV-2 between humans and cats. Furthermore, the reported mutations could affect the viral evasion from immune response, the viral infectivity and pathogenicity. Hence, regular complete genome sequencing of SARS-CoV-2 in humans and nearby companion animals, as well as studying the recombination events in such viruses, are recommended in order to track newly emerging mutations and investigate their effects on viral adaptation, transmission, and their impact on therapeutics and vaccine design.

## Data Availability Statement

The original contributions presented in the study are included in the article/**Supplementary Material**. Further inquiries can be directed to the corresponding author.

## Ethics Statement

The study was conducted according to ethical guidelines and approved by the Ethics Committee of the Animal Health Research Institute (protocol code 11429 and date of approval 12/2020). The patients/participants provided their written informed consent to participate in this study. Written informed consent was obtained from the owners for the participation of their animals in this study. Written informed consent was obtained from the individual(s) for the publication of any potentially identifiable images or data included in this article.

## Author Contributions

Conceptualization, MH, AE-D, NH, MS, and HH; methodology, MH, AE-D, NH, MS, and HH; software, MH and OA; validation, MH, AE-D, NH, MS, OA, and HH; formal analysis, MH, OA, and HH; investigation, MH, AE-D, NH, MS, OA, and HH; resources, MH, AE-D, NH, MS, OA, and HH; data curation, MH, AE-D, NH, OA, and HH; writing—original draft preparation, MH, OA, and HH; writing—review and editing, MH, OA, and HH; visualization, MH and OA; supervision, AE-D, NH, MS, and HH; project administration, MH, NH, and HH; funding acquisition, MH. All authors have read and agreed to the published version of the manuscript.

## Funding

This research was funded by the Science, Technology & Innovation Funding Authority (STDFA), grant number 44585.

## Conflict of Interest

The authors declare that the research was conducted in the absence of any commercial or financial relationships that could be construed as a potential conflict of interest.

## Publisher’s Note

All claims expressed in this article are solely those of the authors and do not necessarily represent those of their affiliated organizations, or those of the publisher, the editors and the reviewers. Any product that may be evaluated in this article, or claim that may be made by its manufacturer, is not guaranteed or endorsed by the publisher.

## References

[B1] Alanagreh L. A. Ababneh M. Al-Shudifat A.-E. Ajlouny M. Abu-Shaikh H. Alzoughool F. (2021). New SARS-CoV-2 Variant From Jordan. Microbiol. Resour. Announc. 10 (26), e00532–e00521. doi: 10.1128/MRA.00532-21 PMC8248861 34197207

[B2] Alghoribi M. F. Alswaji A. Okdah L. Alhayli S. Zinab B. Alzayer M. A.. (2021). Emergence of New SARS-CoV-2 Variant Under Investigation in Saudi Arabia. doi: 10.21203/rs.3.rs-847384/v1

[B3] Altschul S. F. Gish W. Miller W. Myers E. W. Lipman D. J. (1990). Basic Local Alignment Search Tool. J. Mol. Biol. 215 (3), 403–410. doi: 10.1016/S0022-2836(05)80360-2 2231712

[B4] Begum F. Mukherjee D. Das S. Thagriki D. Tripathi P. P. Banerjee A. K.. (2020). Specific Mutations in SARS-CoV2 RNA Dependent RNA Polymerase and Helicase Alter Protein Structure, Dynamics and Thus Function: Effect on Viral RNA Replication. BioRxiv. doi: 10.1101/2020.04.26.063024

[B5] Biorender (2021). Available at: https://app.biorender.com/.

[B6] Bolles M. Donaldson E. Baric R. (2011). Sars-CoV and Emergent Coronaviruses: Viral Determinants of Interspecies Transmission. Curr. Opin. Virol. 1 (6), 624–634. doi: 10.1016/j.coviro.2011.10.012 22180768 PMC3237677

[B7] Bosco-Lauth A. M. Hartwig A. E. Porter S. M. Gordy P. W. Nehring M. Byas A. D.. (2020). Experimental Infection of Domestic Dogs and Cats With SARS-CoV-2: Pathogenesis, Transmission, and Response to Reexposure in Cats. Proc. Natl. Acad. Sci. 117 (42), 26382–26388. doi: 10.1073/pnas.2013102117 32994343 PMC7585007

[B8] Cai Y. Zhang J. Xiao T. Peng H. Sterling S. M. Walsh R. M.. (2020). Distinct Conformational States of SARS-CoV-2 Spike Protein. Science 369 (6511), 1586–1592. doi: 10.1126/science.abd4251 32694201 PMC7464562

[B9] Calvet G. A. Pereira S. A. Ogrzewalska M. Pauvolid-Corrêa A. Resende P. C. Tassinari W. D. S.. (2021). Investigation of SARS-CoV-2 Infection in Dogs and Cats of Humans Diagnosed With COVID-19 in Rio De Janeiro, Brazil. PloS One 16 (4), e0250853. doi: 10.1371/journal.pone.0250853 33909706 PMC8081175

[B10] Castelli M. Baj A. Criscuolo E. Ferrarese R. Diotti R. A. Sampaolo M.. (2021). Characterization of a Lineage C. 36 SARS-CoV-2 Isolate With Reduced Susceptibility to Neutralization Circulating in Lombardy, Italy. Viruses 13 (8), 1514. doi: 10.3390/v13081514 34452380 PMC8402759

[B11] Chan J. F.-W. Kok K. -H. Zhu Z. Chu H. To K.K. -W. Yuan S.. (2020). Genomic Characterization of the 2019 Novel Human-Pathogenic Coronavirus Isolated From a Patient With Atypical Pneumonia After Visiting Wuhan. Emerg. Microbes Infect. 9 (1), 221–236. doi: 10.1080/22221751.2020.1719902 31987001 PMC7067204

[B12] Chaw S. -M. Tai J. -H. Chen S. -L. Hsieh C. -H. Chang S. -Y. Yeh S. -H.. (2020). The Origin and Underlying Driving Forces of the SARS-CoV-2 Outbreak. J. Biomed. Sci. 27, 1–12. doi: 10.1186/s12929-020-00665-8 32507105 PMC7276232

[B13] Chen J. Wang R. Wang M. Wei G.-W. (2020). Mutations Strengthened SARS-CoV-2 Infectivity. J. Mol. Biol. 432 (19), 5212–5226. doi: 10.1016/j.jmb.2020.07.009 32710986 PMC7375973

[B14] Crooks G. E. Hon G. Chandonia J.-M. Brenner S. E. (2004). WebLogo: A Sequence Logo Generator. Genome Res. 14 (6), 1188–1190. doi: 10.1101/gr.849004 15173120 PMC419797

[B15] Deng X. Garcia-Knight M. A. Khalid M. M. Servellita V. Wang C. Morris M. K.. (2021). Transmission, Infectivity, and Antibody Neutralization of an Emerging SARS-CoV-2 Variant in California Carrying a L452R Spike Protein Mutation. medRxiv. doi: 10.1101/2021.03.07.21252647

[B16] Duan L. Zheng Q. Zhang H. Niu Y. Lou Y. Wang H. (2020). The SARS-CoV-2 Spike Glycoprotein Biosynthesis, Structure, Function, and Antigenicity: Implications for the Design of Spike-Based Vaccine Immunogens. Front. Immunol. 11, 2593. doi: 10.3389/fimmu.2020.576622 PMC7575906 33117378

[B17] Duffy S. (2018). Why are RNA Virus Mutation Rates So Damn High? PloS Biol. 16 (8), e3000003. doi: 10.1371/journal.pbio.3000003 30102691 PMC6107253

[B18] Eastman P. Swails J. Chodera J. D. Mcgibbon R. T. Zhao Y. Beauchamp K. A.. OpenMM 7: Rapid Development of High Performance Algorithms for Molecular Dynamics 13, 7, e1005659. doi: 10.1371/journal.pcbi.1005659 PMC5549999 28746339

[B19] Edgar R. C. (2004). MUSCLE: Multiple Sequence Alignment With High Accuracy and High Throughput. Nucleic Acids Res. 32 (5), 1792–1797. doi: 10.1093/nar/gkh340 15034147 PMC390337

[B20] Elaswad A. Fawzy M. Basiouni S. Shehata A. A. (2020). Mutational Spectra of SARS-CoV-2 Isolated From Animals. PeerJ 8, e10609. doi: 10.7717/peerj.10609 33384909 PMC7751428

[B21] Eswar N. John B. Mirkovic N. Fiser A. Ilyin V. A. Pieper U.. (2003). Tools for Comparative Protein Structure Modeling and Analysis. Nucleic Acids Res. 31 (13), 3375–3380. doi: 10.1093/nar/gkg543 12824331 PMC168950

[B22] Fung T. S. Liu D. X. (2019). Human Coronavirus: Host-Pathogen Interaction. Annu. Rev. Microbiol. 73, 529–557. doi: 10.1146/annurev-micro-020518-115759 31226023

[B23] Guruprasad L. (2021). Human SARS CoV-2 Spike Protein Mutations. Proteins: Struct. Function Bioinf. 89 (5), 569–576. doi: 10.1002/prot.26042 PMC8014176 33423311

[B24] Hall T. (1999). BioEdit: A User-Friendly Biological Sequence Alignment Editor and Analysis Program for Windows 95/98/Nt. Nucleic Acids Symp. Ser. 41, 95–8.

[B25] Hamer S. A. Pauvolid-Corrêa A. Zecca I. B. Davila E. Auckland L. D. Roundy C. M.. (2021). SARS-Cov-2 Infections and Viral Isolations Among Serially Tested Cats and Dogs in Households With Infected Owners in Texas, Usa. Viruses 13 (5), 938. doi: 10.3390/v13050938 34069453 PMC8159091

[B26] Hamming I. Timens W. Bulthuis M. Lely A. Navis G. V. Van Goor H.. (2004). Tissue Distribution of ACE2 Protein, the Functional Receptor for SARS Coronavirus. A First Step in Understanding SARS Pathogenesis. J. Pathol.: A J. Pathol. Soc. Great Britain Ireland 203 (2), 631–637. doi: 10.1002/path.1570 PMC7167720 15141377

[B27] Harvey W. T. Carabelli A. M. Jackson B. Gupta R. K. Thomson E. C. Harrison E. M.. (2021). Sars-CoV-2 Variants, Spike Mutations and Immune Escape. Nat. Rev. Microbiol. 19 (7), 409–424. doi: 10.1038/s41579-021-00573-0 34075212 PMC8167834

[B28] Henderson R. Edwards R. J. Mansouri K. Janowska K. Stalls V. Gobeil S. M.. (2020). Controlling the SARS-CoV-2 Spike Glycoprotein Conformation. Nat. Struct. Mol. Biol. 27 (10), 925–933. doi: 10.1038/s41594-020-0479-4 32699321 PMC8581954

[B29] Hobbs E. C. Reid T. J. (2021). Animals and SARS-CoV-2: Species Susceptibility and Viral Transmission in Experimental and Natural Conditions, and the Potential Implications for Community Transmission. Transbound. Emerg. Dis. 68 (4), 1850–1867. doi: 10.1111/tbed.13885 33091230 PMC8359434

[B30] Hodcroft E. B. Domman D. B. Snyder D. J. Oguntuyo K. Van Diest M. Densmore K. H.. (2021). Emergence in Late 2020 of Multiple Lineages of SARS-CoV-2 Spike Protein Variants Affecting Amino Acid Position 677. MedRxiv. doi: 10.1101/2021.02.12.21251658

[B31] Hoffmann M. Kleine-Weber H. Schroeder S. Krüger N. Herrler T. Erichsen S.. (2020). Sars-CoV-2 Cell Entry Depends on ACE2 and TMPRSS2 and is Blocked by a Clinically Proven Protease Inhibitor. Cell 181 (2), 271–280.e8. doi: 10.1016/j.cell.2020.02.052 32142651 PMC7102627

[B32] Jones D. T. Taylor W. R. Thornton J. M. (1992). The Rapid Generation of Mutation Data Matrices From Protein Sequences. Bioinformatics 8 (3), 275–282. doi: 10.1093/bioinformatics/8.3.275 1633570

[B33] Korber B. Fischer W. Gnanakaran S. Yoon H. Theiler J. Abfalterer W.. (2020). Tracking Changes in SARS-CoV-2 Spike: Evidence That D614G Increases Infectivity of the COVID-19 Virus. Cell 182 (4), 812–827.e19. doi: 10.1016/j.cell.2020.06.043 32697968 PMC7332439

[B34] Korber B. Fischer W. Gnanakaran S. Yoon H. Theiler J. Abfalterer W. (2020b). Spike Mutation Pipeline Reveals the Emergence of a More Transmissible Form of SARS-Cov-2. bioRxiv. doi: 10.1101/2020.04.29.069054

[B35] Kubik S. Arrigo N. Bonet J. Xu Z.. (2021). Mutational Hotspot in the SARS-CoV-2 Spike Protein N-terminal Domain Conferring Immune Escape Potential. Viruses 13, 2114–2126. 34834921 10.3390/v13112114 PMC8618472

[B36] Kumar S. Stecher G. Tamura K. (2016). MEGA7: Molecular Evolutionary Genetics Analysis Version 7.0 for Bigger Datasets. Mol. Biol. Evol. 33 (7), 1870–1874. doi: 10.1093/molbev/msw054 27004904 PMC8210823

[B37] Lauring A. S. Hodcroft E. B. (2021). Genetic Variants of SARS-CoV-2—What do They Mean? JAMA. doi: 10.1001/jama.2020.27124 33404586

[B38] Liu Z. Vanblargan L. A. Bloyet L. -M. Rothlauf P. W. Chen R. E. Stumpf S.. (2021). Identification of SARS-CoV-2 Spike Mutations That Attenuate Monoclonal and Serum Antibody Neutralization. Cell Host Microbe 29 (3), 477–488.e4. doi: 10.1016/j.chom.2021.01.014 33535027 PMC7839837

[B39] Luan J. Lu Y. Jin X. Zhang L.. (2020). Spike Protein Recognition of Mammalian ACE2 Predicts the Host Range and an Optimized ACE2 for SARS-CoV-2 Infection. Biochem. Biophys. Res. Commun. 526 (1), 165–169. doi: 10.1016/j.bbrc.2020.03.047 32201080 PMC7102515

[B40] Lu G. Wang Q. Gao G. F. (2020). Genomic Characterisation and Epidemiology of 2019 Novel Coronavirus: Implications for Virus Origins and Receptor Binding. Trends Microbiol. 23, 468–78. doi: 10.1016/j.tim.2015.06.003 PMC7159086 32007145

[B41] Lu R. Zhao X. Li J. Niu P. Yang B. Wu H. (2015). Bat-to-Human: Spike Features Determining ‘Host Jump’of Coronaviruses SARS-CoV, Mers-CoV, and Beyond. Lancet 395 (10224), 565–574. doi: 10.1016/S0140-6736(20)30251-8 PMC7125587 26206723

[B42] Maier J. A. Martinez C. Kasavajhala K. Wickstrom L. Hauser K. E. Simmerling C.. (2015). ff14SB: Improving the Accuracy of Protein Side Chain and Backbone Parameters From Ff99sb. J. Chem. Theory Comput. 11 (8), 3696–3713. doi: 10.1021/acs.jctc.5b00255 26574453 PMC4821407

[B43] Mccallum M. Bassi J. De Marco A. Chen A. Walls A. C. Di Iulio J.. (2021). SARS-CoV-2 Immune Evasion by the B. 1.427/B. 1.429 Variant of Concern. Science 373 (6555), 648–654. doi: 10.1126/science.abi7994. 34210893 PMC9835956

[B44] Mervat Hamdy A. E. Hagag N. Liyanage N. Shaheen M. Ahmed H. (2021). SARS-CoV-2 Infection of Companion Animals in Some Egyptian Governorates: Risk of Spill Over and Reverse Zoonoses. Submitted Publ. Vet. Med. Sci.

[B45] Naqvi A. a. T. Fatima K. Mohammad T. Fatima U. Singh I. K. Singh A.. (2020). Insights Into SARS-CoV-2 Genome, Structure, Evolution, Pathogenesis and Therapies: Structural Genomics Approach. Biochim. Biophys. Acta (BBA) Molecular Basis Dis. 1866 (10), 165878. doi: 10.1016/j.bbadis.2020.165878 PMC7293463 32544429

[B46] NCBI (2021) Refseq of SARS-CoV-2 Spike Gene. Available at: https://www.ncbi.nlm.nih.gov/gene/43740568.

[B47] Newman A. Smith D. Ghai R. R. Wallace R. M. Torchetti M. K. Loiacono C.. (2020). First Reported Cases of SARS-CoV-2 Infection in Companion Animals—New York, March–April 2020. Morbid. Mortal. Weekly Rep. 69 (23), 710. doi: 10.15585/mmwr.mm6923e3 PMC7315787 32525853

[B48] OIE (2021) Sars-COV-2 in Animals Situation Report. Available at: https://www.oie.int/app/uploads/2021/10/sars-cov-2-situation-report-5.pdf.

[B49] Ortega J. T. Serrano M. L. Pujol F. H. Rangel H. R. (2020). Role of Changes in SARS-CoV-2 Spike Protein in the Interaction With the Human ACE2 Receptor: An in Silico Analysis. EXCLI J. 19, 410. doi: 10.3390/pathogens11010045 32210742 PMC7081066

[B50] Papadopoulos J. S. Agarwala R. (2007). COBALT: Constraint-Based Alignment Tool for Multiple Protein Sequences. Bioinformatics 23 (9), 1073–1079. doi: 10.1093/bioinformatics/btm076 17332019

[B51] Pater A. A. Bosmeny M. S. Barkau C. L. Ovington K. N. Chilamkurthy R. Parasrampuria M.. (2021). Emergence and Evolution of a Prevalent New SARS-CoV-2 Variant in the United States. bioRxiv. doi: 10.1101/2021.01.11.426287

[B52] Patterson E. I. Elia G. Grassi A. Giordano A. Desario C. Medardo M.. (2020). Evidence of Exposure to SARS-CoV-2 in Cats and Dogs From Households in Italy. Nat. Commun. 11 (1), 1–5. doi: 10.1038/s41467-020-20097-0 33277505 PMC7718263

[B53] Pettersen E. F. Goddard T. D. Huang C. C. Couch G. S. Greenblatt D. M. Meng E. C.. (2004). Ucsf Chimera—a Visualization System for Exploratory Research and Analysis. J. Comput. Chem. 25 (13), 1605–1612. doi: 10.1002/jcc.20084 15264254

[B54] Plante J. A. Liu Y. Liu J. Xia H. Johnson B. A. Lokugamage K. G.. (2021). Spike Mutation D614G Alters SARS-CoV-2 Fitness. Nature 592 (7852), 116–121. doi: 10.1038/s41586-020-2895-3 33106671 PMC8158177

[B55] Prince T. Smith S. L. Radford A. D. Solomon T. Hughes G. L. Patterson E. I.. (2021). SARS-Cov-2 Infections in Animals: Reservoirs for Reverse Zonosis and Models for Study. Viruses 13 (3), 494. doi: 10.3390/v13030494 33802857 PMC8002747

[B56] Ren L.-L. Wang Y.-M. Wu Z.-Q. Xiang Z.-C. Guo L. Xu T.. (2020). Identification of a Novel Coronavirus Causing Severe Pneumonia in Human: A Descriptive Study. Chin. Med. J. doi: 10.1097/CM9.0000000000000722 PMC7147275 32004165

[B57] Rice P. Longden I. Bleasby A. (2000). EMBOSS: The European Molecular Biology Open Software Suite. Trends Genet. 16 (6), 276–277. doi: 10.1016/S0168-9525(00)02024-2 10827456

[B58] Ruiz‐Arrondo I. Portillo A. Palomar A. M. Santib´ñez S. Santib´ñez P. Cervera C.. (2021). Detection of SARS-CoV-2 in Pets Living With COVID-19 Owners Diagnosed During the COVID-19 Lockdown in Spain: A Case of an Asymptomatic Cat With SARS-CoV-2 in Europe. Transbound. Emerg. Dis. 68 (2), 973–976. doi: 10.1111/tbed.13803 32810370 PMC7461521

[B59] Sailleau C. Dumarest M. Vanhomwegen J. Delaplace M. Caro V. Kwasiborski A.. (2020). First Detection and Genome Sequencing of SARS-CoV-2 in an Infected Cat in France. Transbound. Emerg. Dis. 67 (6), 2324–2328. doi: 10.1111/tbed.13659 32500944 PMC7300955

[B60] Schneider T. D. Stephens R. M. (1990). Sequence Logos: A New Way to Display Consensus Sequences. Nucleic Acids Res. 18 (20), 6097–6100. doi: 10.1093/nar/18.20.6097 2172928 PMC332411

[B61] Shafique L. Ihsan A. Liu Q. (2020). Evolutionary Trajectory for the Emergence of Novel Coronavirus SARS-Cov-2. Pathogens 9 (3), 240. doi: 10.3390/pathogens9030240 PMC7157669 32210130

[B62] Shang J. Wan Y. Luo C. Ye G. Geng Q. Auerbach A.. (2020). Cell Entry Mechanisms of SARS-Cov-2. Proc. Natl. Acad. Sci. 117 (21), 11727–11734. doi: 10.1073/pnas.2003138117 32376634 PMC7260975

[B63] Shi J. Wen Z. Zhong G. Yang H. Wang C. Huang B.. (2020). Susceptibility of Ferrets, Cats, Dogs, and Other Domesticated Animals to SARS–coronavirus 2. Science 368 (6494), 1016–1020. doi: 10.1126/science.abb7015 32269068 PMC7164390

[B64] Sophiagenetics (2021) Sophiagenetics DDM Database of SARS-Cov-2. Available at: https://www.sophiagenetics.com/hospitals/solutions/sars-cov-2/.

[B65] Su S. Wong G. Shi W. Liu J. Lai A. C. Zhou J.. (2016). Epidemiology, Genetic Recombination, and Pathogenesis of Coronaviruses. Trends Microbiol. 24 (6), 490–502. doi: 10.1016/j.tim.2016.03.003 27012512 PMC7125511

[B66] Walls A. C. Tortorici M. A. Bosch B. -J. Frenz B. Rottier P.J. Dimaio F.. (2016). Cryo-Electron Microscopy Structure of a Coronavirus Spike Glycoprotein Trimer. Nature 531 (7592), 114–117. doi: 10.2210/pdb3jcl/pdb 26855426 PMC5018210

[B67] Wan Y. Shang J. Graham R. Baric R. S. Li F. (2020). Receptor Recognition by the Novel Coronavirus From Wuhan: An Analysis Based on Decade-Long Structural Studies of SARS Coronavirus. J. Virol. 94 (7), e00127–e00120. doi: 10.1128/JVI.00127-20 31996437 PMC7081895

[B68] Wang Q. Zhang Y. Wu L. Niu S. Song C. Zhang Z.. (2020). Structural and Functional basis of SARS-CoV-2 Entry by Using Human ACE2. Cell 181 (4), 894–904.e899. doi: 10.1016/j.cell.2020.03.045 32275855 PMC7144619

[B69] Yan R.. (2020). Structural Basis for the Recognition of SARS-CoV-2 by Full-Length Human ACE2. Science 367 (6485), 1444–1448. doi: 10.1126/science.abb2762 32132184 PMC7164635

[B70] Ye Z.-W. Yuan S. Yuen K.-S. Fung S.-Y. Chan C.-P. Jin D.-Y. (2020). Zoonotic Origins of Human Coronaviruses. Int. J. Biol. Sci. 16 (10), 1686. doi: 10.7150/ijbs.45472 32226286 PMC7098031

[B71] Yurkovetskiy L. Wang X. Pascal K. E. Tomkins-Tinch C. Nyalile T. P. Wang Y.. (2020). Structural and Functional Analysis of the D614G Sars-CoV-2 Spike Protein Variant. Cell 183 (3), 739–751.e8. doi: 10.1016/j.cell.2020.09.032 32991842 PMC7492024

[B72] Zhang J. Cai Y. Xiao T. Lu J. Peng H. Sterling S.M.. (2021). Structural Impact on SARS-CoV-2 Spike Protein by D614G Substitution. Science 372 (6541), 525–530. doi: 10.1126/science.abf2303 33727252 PMC8139424

[B73] Zhang W. Davis B. D. Chen S. S. Martinez J. M. S. Plummer J. T. Vail E.. (2021). Emergence of a Novel SARS-CoV-2 Variant in Southern California. Jama 325 (13), 1324–1326. doi: 10.1001/jama.2021.1612 33571356 PMC7879386

[B74] Zhou P. Yang X. -L. Wang X. -G. Hu B. Zhang L. Zhang W.. (2020). A Pneumonia Outbreak Associated With a New Coronavirus of Probable Bat Origin. Nature 579 (7798), 270–273. doi: 10.1038/s41586-020-2012-7 32015507 PMC7095418

