# Supplementary material for: Mutations of the SARS-CoV-2 Spike Glycoprotein Detected in Cats and Their Effect on Its Structure and Function
Source: Front Cell Infect Microbiol. 2022 Jun 1;12:875123. doi: 10.3389/fcimb.2022.875123 (PMC9198574; doi:10.3389/fcimb.2022.875123)
Supplement: Supplementary file 1 [file DataSheet_1.docx]

**Mutations of the SARS-CoV-2 spike glycoprotein detected in cats and their effect on its structure and function**

**Mervat E. Hamdy^1^, Ayman H. El‑Deeb^2,3^, Naglaa M. Hagag^1^, Momtaz A. Shahein^4^, Osama Alaidi^5,6^ and Hussein A. Hussein^2*^**

^1^ Genome Research Unit, Animal Health Research Institute, Agriculture Research Centre, Giza 12618, Egypt

^2^ Department of Virology, Faculty of Veterinary Medicine, Cairo University, Giza 12211, Egypt

^3^ Department of Virology, Faculty of Veterinary Medicine, King Salman International University, South Sinai, Egypt

^4^ Department of Virology, Animal Health Research Institute, Agriculture Research Centre, Giza 12618, Egypt

^5^ Department of Research and Development, Biocomplexity for Research and Consulting, Cairo, Egypt

^6^ Department of Pharmaceutical Sciences, University of Tennessee Health Science Center, Memphis, Tennessee, 38163, United States.

^*^Corresponding author: Hussein A. Ahmed; Email: husvirol@cu.edu.eg; Department of Virology, Faculty of Veterinary Medicine, Cairo University, Giza 12211, Egypt

**Supplementary Materials:** The following are available online at www.mdpi.com/xxx/s1,

**Supplementary Table S1.** The detailed molecular structure of the spike gene with positions and lengths of its various parts, residue numbering is according to the Wuhan strain (Refseq NC_045512.2, Gene ID: 43740568) ([Guruprasad, 2021](#_ENREF_1);[NCBI, 2021](#_ENREF_2)).

**Supplementary Table S2.** Full Spike gene-specific primers used for conventional RT-PCR and sequencing as described previously ([Ren et al., 2020](#_ENREF_3)).

**Supplementary Table S3.** Case history of companion cats and their owners infected with SARS-CoV-2 and the veterinarian in contact with the infected cats with cycle threoshold (CT) values by real-time RT-PCR.

**Supplementary Table S4.** Identity matrix of the sequenced samples along with some circulating SARS-CoV-2 variants**.**

**Supplementary Table S5.** Identity matrix of the aligned selected sequences residues present at the binding interface between the ACE2 and the viral RBD in different species.

**Supplementary Figure S1.** The impact of the A570V mutation of the spike protein.

**Supplementary Figure S2.** Predicted RBD with the L452R mutation in complex with the human receptor and the RBD-receptor complexes in various other hosts.

**Supplementary Table S1.** The detailed molecular structure of the spike gene with positions and lengths of its various parts, residue numbering is according to the Wuhan strain (Refseq NC_045512.2, Gene ID: 43740568) ([Guruprasad, 2021](#_ENREF_1);[NCBI, 2021](#_ENREF_2)).

|  | Region | Name | Location | Length |
| --- | --- | --- | --- | --- |
| 1 | Subunit 1 (S1) | N-terminal domain of the S1 subunit (NTD) | 13..304 | 292 aa |
| 2 |  | Receptor-Binding Domain (RBD) | 319..528 | 210 aa |
| 3 |  | Receptor binding motif (RBM) | 438..508 | 71 aa |
| 4 |  | C- terminal domains 1 (CTD1) | 528..591 | 64 aa |
| 5 |  | C- terminal domains 2 (CTD2) | 592..686 | 96 aa |
| 6 | S1/S2 cleavage region | S1/S2 cleavage region | 672..709 | 38 aa |
| 7 | Subunit 2 (S2) | Fusion peptide (FP) | 816..833 | 18 aa |
| 8 |  | Fusion-peptide proximal region (FPPR) | 834..910 | 77 aa |
| 9 |  | Heptad repeat 1 (HR1) | 911..985 | 76 aa |
| 10 |  | Central helix (CH) | 985..1035 | 51 aa |
| 11 |  | β-hairpin | 1036..1068 | 34 aa |
| 12 |  | Heptad repeat 2 (HR2) | 1163..1211 | 49 aa |
| 13 |  | Transmembrane region (TM) | 1212..1234 | 24 aa |
| 14 |  | Intracellular region (IC) | 1235..1273 | 41 aa |

**Supplementary Table S2.** Full Spike gene-specific primers used for conventional RT-PCR and sequencing as described previously ([Ren et al., 2020](#_ENREF_3)).

| No | Sequence (5'-3') | Start | Stop | Gene region & Product size | Reference |
| --- | --- | --- | --- | --- | --- |
| 1 | F: CTTGGAGGTTCCGTGGCTAT | 21145 | 21164 | S (1076 bp) | ([Ren et al., 2020](#_ENREF_3)) |
|  | R: AAACCCTGAGGGAGATCACG | 22221 | 22202 |  |  |
| 2 | F: TATCTTGGCAAACCACGCGA | 21289 | 21308 | S (1056 bp) |  |
|  | R: ACCAGCTGTCCAACCTGAAG | 22345 | 22326 |  |  |
| 3 | F: CCCTCAGGGTTTTTCGGCTT | 22210 | 22229 | S (1092 bp) |  |
|  | R: CTGTGGATCACGGACAGCAT | 23302 | 23283 |  |  |
| 4 | F: CCAGCAACTGTTTGTGGACC | 23123 | 23142 | S (1026 bp) |  |
|  | R: GTGGCAAAACAGTAAGGCCG | 24149 | 24130 |  |  |
| 5 | F: ACTTGCAGATGCTGGCTTCA | 24043 | 24062 | S (1083 bp) |  |
|  | R: CTCATTGAGGCGGTCAATTTCT | 25126 | 25105 |  |  |
| 6 | F: TGATTTAGGTGACATCTCTGGCA | 25054 | 25076 | S (1105 bp) |  |
|  | R: ACAACTCCGGATGAACCGTC | 26159 | 26140 |  |  |
| 7 | F: GCTGGCTTGATTGCCATAGT | 25226 | 25245 | S, ORF3 (1136 bp) |  |
|  | R: ACAATCGAAGCGCAGTAAGG | 26362 | 26343 |  |  |

**Supplementary Table S3.** Case history of companion cats and their owners infected with SARS-CoV-2 and the veterinarian in contact with the infected cats with cycle threoshold (CT) values by real-time RT-PCR.

| **Sample ID** | **Location/date** | **Species** | **Case history of owner** | **Case history of pet animal** | **CT of rRT-PCR of the positive samples** | |
| --- | --- | --- | --- | --- | --- | --- |
|  |  |  |  |  | **N gene** | **ORF 1ab gene** |
| Sample 1 | Clinic/December 2020 | Cat | 20 years old, female, COVID-19 positive with severe respiratory signs | 2 years cat, severe respiratory signs, vomiting | 30.152 | 30.543 |
| Sample 2 | Clinic/March 2021 | Cat | 31 years old, female, COVID-19 positive with severe respiratory signs | 1.5 years, cat, otitis and severe respiratory signs. | 30.152 | 31.094 |
| Sample 3 | Clinic/July 2021 | Cat | 41 years old, female, COVID-19 positive with severe respiratory signs | 15 day kitten, severe respiratory signs, died in July 2021 | 27.56 | 28.32 |
| Sample 4 | Clinic/July 2021 | Cat |  | 3 years cat, the mother of the previous 15 day kitten, asymptomatic | 28.510 | 29.358 |
| Sample 5 | Clinic/July 2021 | Human | 28 years old, male, asymptomatic veterinarian who dealing with positive COVID-19 cats and the dead kitten. | | 30.996 | 31.387 |

**Supplementary Table S4.** Identity matrix of the sequenced samples along with some circulating SARS-CoV-2 variants**.**

|  |  | 1 | 2 | 3 | 4 | 5 | 6 | 7 | 8 | 9 | 10 | 11 | 12 |
| --- | --- | --- | --- | --- | --- | --- | --- | --- | --- | --- | --- | --- | --- |
| 1 | hCoV-19/Cat/Egypt/AHRI/December/2020(OK144251) | ID | 99.90% | 99.70% | 99.70% | 99.70% | 99.90% | 99.70% | 99.50% | 100.00% | 99.90% | 99.60% | 99.80% |
| 2 | hCoV-19/Cat/Egypt/AHRI/March/2021(OK144252) | 99.90% | ID | 99.70% | 99.70% | 99.70% | 99.90% | 99.70% | 99.60% | 99.90% | 100.00% | 99.60% | 99.80% |
| 3 | hCoV-19/Cat-Mother/Egypt/AHRI/July/2021(OK144253) | 99.70% | 99.70% | ID | 100.00% | 100.00% | 99.60% | 99.90% | 99.50% | 99.70% | 99.70% | 99.60% | 99.60% |
| 4 | hCoV-19/Cat-Kitten/Egypt/AHRI/July/2021(OK144254) | 99.70% | 99.70% | 100.00% | ID | 100.00% | 99.60% | 99.90% | 99.50% | 99.70% | 99.70% | 99.60% | 99.60% |
| 5 | hCoV-19/Veterinarian/Egypt/AHRI/July/2021(OK144255) | 99.70% | 99.70% | 100.00% | 100.00% | ID | 99.60% | 99.90% | 99.50% | 99.70% | 99.70% | 99.60% | 99.60% |
| 6 | NC 045512.2 L/Wuhan-Hu-1 (Reference) | 99.90% | 99.90% | 99.60% | 99.60% | 99.60% | ID | 99.60% | 99.50% | 99.90% | 99.90% | 99.60% | 99.80% |
| 7 | hCoV-19/Egypt/CPHL-S25/2021\|3274157\|2021-07-08 | 99.70% | 99.70% | 99.90% | 99.90% | 99.90% | 99.60% | ID | 99.50% | 99.70% | 99.70% | 99.60% | 99.60% |
| 8 | MZ266636.1 SARS-CoV-2/human/JORDON/AM-HU-16/2021 | 99.50% | 99.60% | 99.50% | 99.50% | 99.50% | 99.50% | 99.50% | ID | 99.50% | 99.60% | 99.20% | 99.50% |
| 9 | Egypt_NRC1_2020\|1315064\|2020-04-26 | 100.00% | 99.90% | 99.70% | 99.70% | 99.70% | 99.90% | 99.70% | 99.50% | ID | 99.90% | 99.60% | 99.80% |
| 10 | Egypt/PHARCO-ARMY-78/03-2021 | 99.90% | 100.00% | 99.70% | 99.70% | 99.70% | 99.90% | 99.70% | 99.60% | 99.90% | ID | 99.60% | 99.80% |
| 11 | hCoV-19/Egypt/CPHL-S26/2021\|3600721\|2021-07-15(Delta)(4.2) | 99.60% | 99.60% | 99.60% | 99.60% | 99.60% | 99.60% | 99.60% | 99.20% | 99.60% | 99.60% | ID | 99.50% |
| 12 | Cat_Italy_VE-IZSVe-20DIA30040-2_2020\|962892\|2020-11-27 | 99.80% | 99.80% | 99.60% | 99.60% | 99.60% | 99.80% | 99.60% | 99.50% | 99.80% | 99.80% | 99.50% | ID |

**Supplementary Table S5.** Identity matrix of the aligned selected sequences residues present at the binding interface between the ACE2 and the viral RBD in different species.

|  |  | 1 | 2 | 3 | 4 | 5 | 6 | 7 | 8 | 9 | 10 |
| --- | --- | --- | --- | --- | --- | --- | --- | --- | --- | --- | --- |
| 1 | NP_001358344.1 ACE 2-isoform 1 precursor [Homo sapiens](Human) | ID | 1 | 1 | 0.746 | 0.788 | 0.838 | 0.795 | 0.781 | 0.774 | 0.647 |
| 2 | XP_018874749.1 ACE 2-[Gorilla gorilla gorilla](Gorilla) | 1 | ID | 1 | 0.746 | 0.788 | 0.838 | 0.795 | 0.781 | 0.774 | 0.647 |
| 3 | XP_016798468.1 ACE 2-isoform X1 [Pan troglodytes](Chimpanzee) | 1 | 1 | ID | 0.746 | 0.788 | 0.838 | 0.795 | 0.781 | 0.774 | 0.647 |
| 4 | XP_032963186.1 ACE 2-[Rhinolophus ferrumequinum](Greater horseshoe bat) | 0.746 | 0.746 | 0.746 | ID | 0.795 | 0.83 | 0.788 | 0.788 | 0.788 | 0.654 |
| 5 | XP_036768816.1 ACE 2-[Manis pentadactyla](Chinese Pangolin) | 0.788 | 0.788 | 0.788 | 0.795 | ID | 0.88 | 0.838 | 0.83 | 0.83 | 0.697 |
| 6 | NP_001034545.1 ACE 2-precursor [Felis catus](Cat) | 0.838 | 0.838 | 0.838 | 0.83 | 0.88 | ID | 0.908 | 0.887 | 0.887 | 0.718 |
| 7 | NP_001158732.1 ACE 2-precursor [Canis lupus familiaris](Domestic Dog) | 0.795 | 0.795 | 0.795 | 0.788 | 0.838 | 0.908 | ID | 0.894 | 0.887 | 0.695 |
| 8 | XP_032187677.1 ACE 2-[Mustela erminea](Mink) | 0.781 | 0.781 | 0.781 | 0.788 | 0.83 | 0.887 | 0.894 | ID | 0.992 | 0.732 |
| 9 | NP_001297119.1 ACE 2-precursor [Mustela putorius furo](Domestic Ferret) | 0.774 | 0.774 | 0.774 | 0.788 | 0.83 | 0.887 | 0.887 | 0.992 | ID | 0.732 |
| 10 | XP_040517014.1 ACE 2-isoform X1 [Gallus gallus](Chicken) | 0.647 | 0.647 | 0.647 | 0.654 | 0.697 | 0.718 | 0.695 | 0.732 | 0.732 | ID |

**
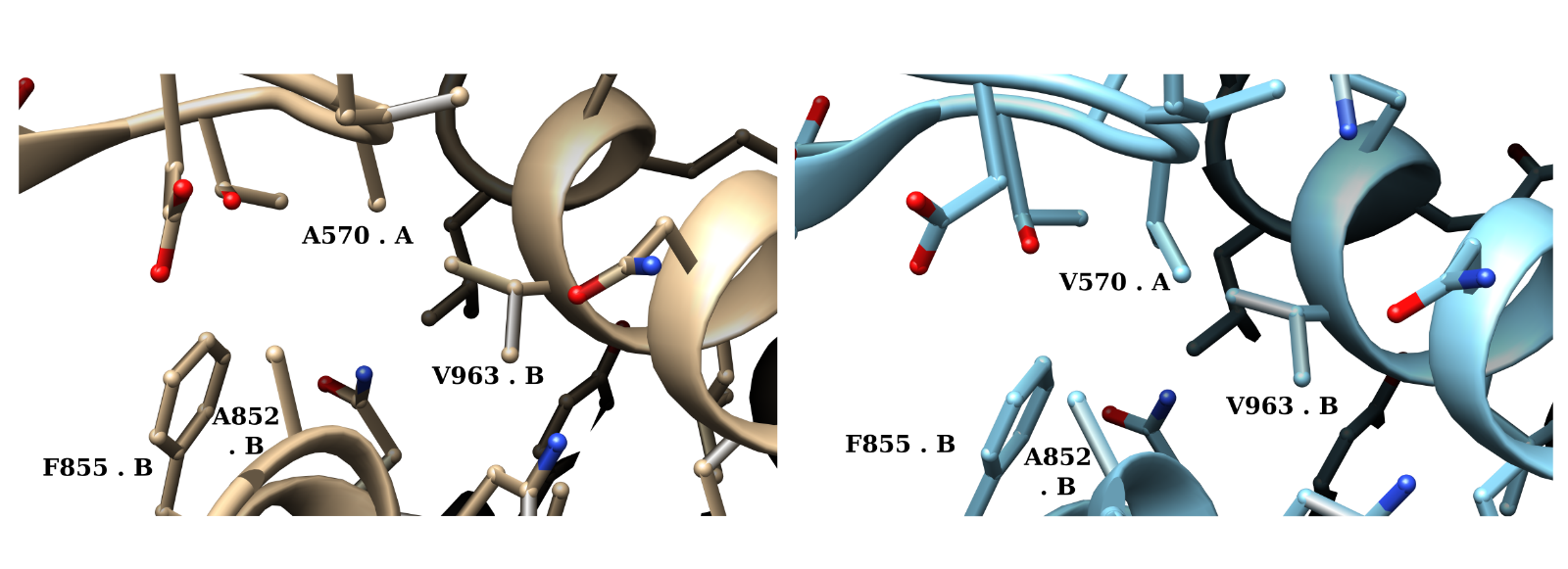
**

**Supplementary Figure S1.** The impact of the A570V mutation of the spike protein.

The figure illustrates a molecular view of the mutation A570V that is found near the surface of the spike protein within the native structure (shown on the left, ribbon is depicted in gold) and the corresponding residues (shown on the right, ribbon is depicted in cyan) in predicted model with the mutant A570V. The residue of intrest and the naghboring hydrophobic residies along with their corresponding chains are labeled (chain names are seprated from the residue numbers with dots). The figure shows that the increase of the hydrophobicity of the residue 570, such as from alanin to valine, is expected to stabilize the interaction of the two naghboring chains within the spike trimer (illustrated here by chains A and B).


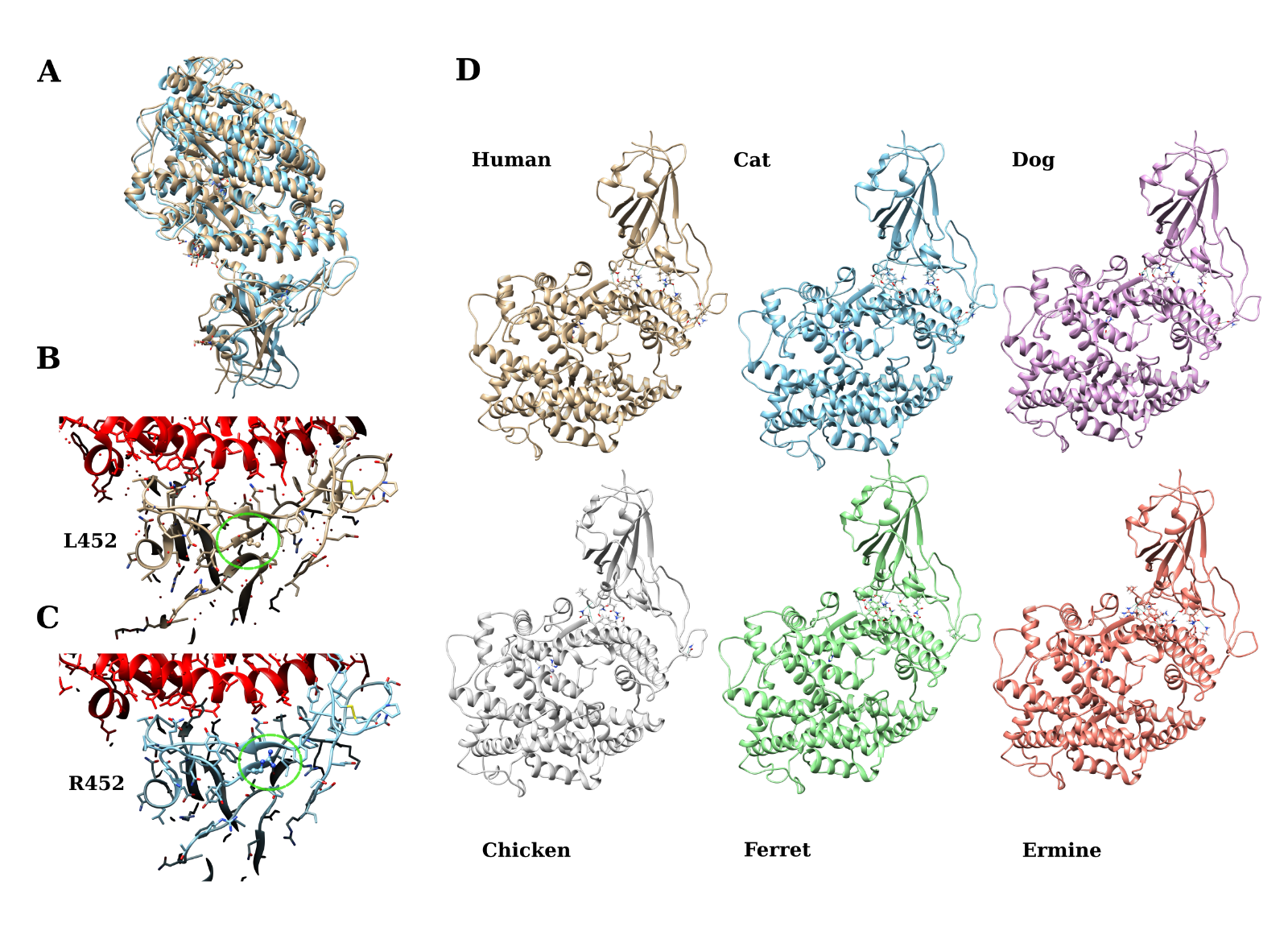


**Supplementary Figure S2.** Predicted RBD with the L452R mutation in complex with the human receptor and the RBD-receptor complexes in various other hosts.

Panel A shows the overall structure of the template versus modelled RBD in complex with the human receptor (depicted in gold and cyan, respectively) in ribbon representation. Panels B and C show the native and predicted RBD in complex with the human receptor, respectively. Both L452 and its corresponding mutant R452 residues (shown in ball and stick representation and surrounded by green circles) are far from the receptor (shown in red colour) interaction surface (panels B and C, respectively). Panel D illustrates the predicted structures of the ACE2-RBD complexes, in the studied animal hosts, shown in ribbon representation. The side chains of amino acids that are interacting (via ploar conatcs) are shown and potential hydrogen bonds are depicted by the blue dashed lines.

Guruprasad, L. (2021). Human SARS CoV‐2 spike protein mutations. *Proteins: Structure, Function, and Bioinformatics* 89**,** 569-576.

Ncbi (2021). *Refseq of SARS-CoV-2 Spike gene* [Online]. National Centre for Biotechnology Information Available: <https://www.ncbi.nlm.nih.gov/gene/43740568> [Accessed].

Ren, L.-L., Wang, Y.-M., Wu, Z.-Q., Xiang, Z.-C., Guo, L., Xu, T., et al. (2020). Identification of a novel coronavirus causing severe pneumonia in human: a descriptive study. *Chinese medical journal*.
